# Bio-Inspired Space Robotic Control Compared to Alternatives

**DOI:** 10.3390/biomimetics9020108

**Published:** 2024-02-12

**Authors:** Timothy Sands

**Affiliations:** 1 Department of Mechanical and Aerospace Engineering, Cornell University, Ithaca, NY 14853, USA; dr.timsands@alumni.stanford.edu; 2 Department of Mechanical and Aerospace Engineering, Naval Postgraduate School, Monterey, CA 93943, USA

**Keywords:** bioinspiration, biomimetics, robotics, control, bio-inspired locomotion, bio-robotics, bio-inspired robots, biomechanics

## Abstract

Controlling robots in space with necessarily low material and structural stiffness is quite challenging at least in part due to the resulting very low structural resonant frequencies or natural vibration. The frequencies are sometimes so low that the very act of controlling the robot with medium or high bandwidth controllers leads to excitation of resonant vibrations in the robot appendages. Biomimetics or biomimicry emulates models, systems, and elements of nature for solving such complex problems. Recent seminal publications have re-introduced the viability of optimal command shaping, and one recent instantiation mimics baseball pitching to propose control of highly flexible space robots. The readership will find a perhaps dizzying array of thirteen decently performing alternatives in the literature but could be left bereft selecting a method(s) deemed to be best suited for a particular application. Bio-inspired control of space robotics is presented in a quite substantial (perhaps not comprehensive) comparison, and the conclusions of this study indicate the three top performing methods based on minimizing control effort (i.e., fuel) usage, tracking error mean, and tracking error deviation, where 96%, 119%, and 80% performance improvement, respectively, are achieved.

## 1. Introduction

**Figure 1:**
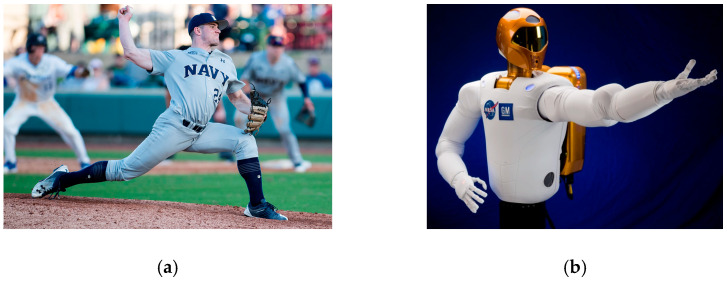
Space robots may be represented as cylindrical center rigid bodies and highly flexible appendages. (**a**) A U.S. Naval Academy pitcher throws to home plate at a baseball tournament (image credit: Technical Sergeant David W. Carbajal) [1,2]. (**b**) NASA’s first humanoid space robot (image credit: NASA) [3,4].

To accumulate energy, baseball pitchers (Figure 1) “wind up”, initially moving the ball in the opposite direction to the desired destination. The general shape of space robots is not dissimilar to baseball pitchers, and this study evaluates the efficacy of trajectory shaping for space robots inspired by the biomimicry of baseball pitching Box 1.

Box 1 **Problem Statement**

**Problem Statement**: Amongst the many available options for autonomously controlling deep space robots, is one method better than another at simultaneously seeking exact precision, vibration elimination, fuel minimization, and robustness?

### 1.1. Broad Context and Why This Study Is Important

**Figure 2:**
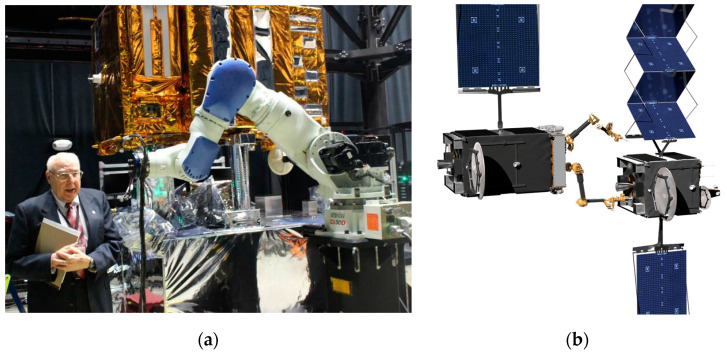
(**a**) NASA mission to repair and refuel satellites on orbit (image credit: NASA [4,10]. (**b**) Satellite servicing mission of the Defense Advanced Research Projects Agency (DARPA). Image credit: DARPA [11,12].

Despite recent demonstrations of operations in space of capabilities at the system level of several autonomous functions [5,6] including robotics, contemporary space operations assessment and action planning rely upon pre-scripted sequences of commands from ground operations personnel [7]. Considering challenging and distant robotics missions (e.g., to Mars), ground operators are unlikely to predict all the likely encounters and physical interactions occurring in parts of space seldomly experienced before [8]. Limited knowledge in complex situations demands autonomy, perceivably establishing a new frontier for space exploration. Another obvious example is the utilization of very small spacecraft in cislunar orbits to refuel, repair, and replenish earth-orbiting spacecraft at lower altitudes, necessitating grappling potentially unknown masses of uncooperative spacecraft. Extreme fuel efficiency seems mandatory, especially due to the low mass and volume of underactuated robotic repair spacecraft [8] like those depicted in Figure 2, leading naturally to control minimization as a primary figure of merit [9].

#### 1.1.1. Cislunar Space

**Figure 3:**
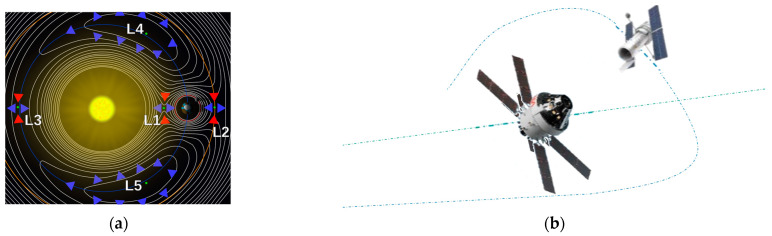
Operations in cislunar orbits **[14]**. (**a**) Schematic defining features of cislunar space. Image credit NASA [15]. (**b**) Cislunar satellite inspector. Image credit: Air Force Research Laboratory and National Aeronautics and Space Administration [2,4].

A recent primer on cislunar space published by the U.S. Air Force [13] intended to aid the development of expertise, capabilities, plans, and operational concepts. The importance of the orbits manifested in the December 2019 creation of a new branch of the military charged with the duties to defend and protect American space interests, especially since highly perturbed orbits depart predictable locations; intercept, rendezvous, and proximity operations (depicted in Figure 3) become quite complicated and potentially unpredictable. Cislunar orbits are generally no longer planar and no longer elliptical (certainly not circular), and spacecraft positions are no longer easy to articulate geometrically.

#### 1.1.2. Cislunar Robotic Operations

**Figure 4:**
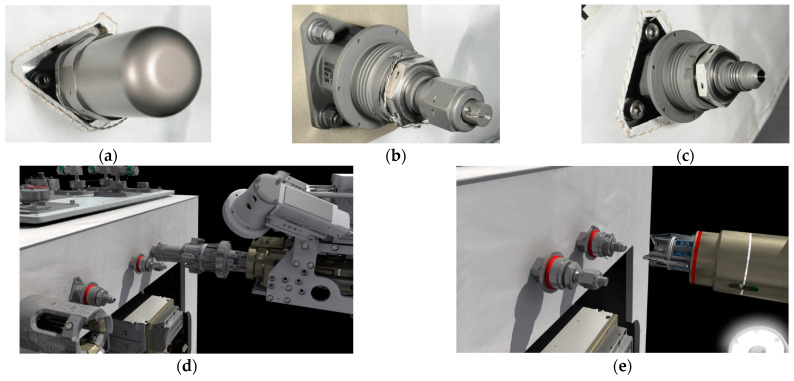
NASA Robotic Refueling Mission (RRM) task: refueling. Individual pieces of hardware show the seals that typical satellite fuel valves have. (**a**) A tertiary cap with a “lock wire” visible underneath; (**b**) a safety cap/actuation nut with a securing lock wire; (**c**) an exposed fuel valve; (**d**) a safety cap tool removing a safety cap; and (**e**) a nozzle tool being connected to the now exposed fuel valve, enabling fuel transfer (image credit: NASA [4,18]).

Actuators for space robots were recently reviewed in [16], highlighting time delays as a key limiting issue for successful operations, some of which are displayed in Figure 4. A disparate review [17] assembled over the last three years subdivided the trends in research development achievements. The reviews focus on two different treatments of modeling uncertainties in hopes of not needing to increase design margins: either increasing the accuracy of parameter discrimination or alternatively developing methods with inherent robustness. The later review [17] emphasized the fact that task performance execution success correlated with onboard computing power.

High-speed maneuvering necessitating the real-time solution of inverse kinematics was proposed in [18,19] for the capture of space debris with safety from possible collisions for dual-arm continuum manipulators with input saturation. Real-time trajectory planning provides an alternative when paired with an adaptive controller using deep reinforcement learning proposed in [19]. Meanwhile, trajectory tracking for attitude maneuvers with vibration suppression was proposed in [20] in the presence of actuator and disturbance uncertainties. Disturbances and actuator saturation was investigated in [21], while compliance control was emphasized in [22], while so–called “soft robotics” was modeled in [23]. Modeling is key, since the robot hardware needs a mathematical model to facilitate experimentation in computers. 

**Figure 5:**
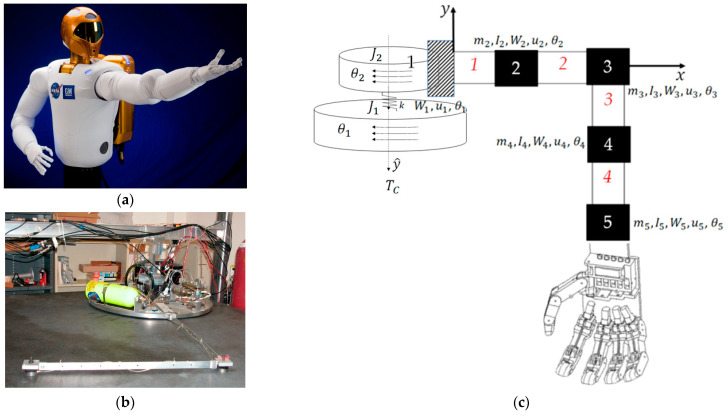
Space robots with cylindrical center rigid bodies and highly flexible appendages. (**a**) NASA’s first humanoid space robot. Image credit: NASA [3]. (**b**) Laboratory flexible rotational spacecraft hub with a free-floating, planar air-bearing, very light robotic arm, the schematic of which is displayed in subfigure (**c**).

In the broadest sense, the space robot (like those depicted in Figure 1, Figure 3 and Figure 5) may be considered generically as a cylindrical main body with a robotic appendage attached, leading to both rigid-body and flexible-body treatments. Figure 5 displays two variations on laboratory hardware replicating space robots. Subfigure (b) is the Navy space robot laboratory hardware that serves as the baseline system analyzed in subsequent sections of this manuscript. The terminology is defined in Table 1 which displays proximal variable definitions and nomenclature. Such convenience to the readership is repeatedly provided (e.g., Table 2, Table 3, Table 4 and Table 5) throughout the manuscript. 

**Table 1:** Table of proximal variables and nomenclature ^1^.

Variable/Acronym	Definition	Variable/Acronym	Definition
$\hat{y}$	Centerline unit vector	k	Appendage stiffness
$T_{C}$	Control torque	$m_{i},I_{i}\forall i=2\ldots 5$	Flexible masses and inertias
$J_{1}$	Main body inertia mass moment	$J_{2}$	Flexible inertia mass moment
$\theta_{1}$	Main body rotation angle	$\theta_{i},W_{i}\forall i=2\ldots 5$	Translation and rotation angles

^1^ Such tables are offered throughout the manuscript to aid readability.

The literature reveals many mathematical approaches [13] for modeling and also for control, while the readership might be bereft and confused about which method or combinations of methods should be considered for their particular application. This manuscript compares a recently proposed bio-inspired approach to many of the contemporary alternatives. 

### 1.2. Broad Review of Modeling and Control from First Principles to Modern Instantiations

Modeling can include external and internal constraints on every particle expressed in a Euclidean space [24] or where Newton’s Laws [25] and Euler’s equation [26] may be combined using Chasle’s theorem [27] to define space robot motion states in six degrees of freedom [28]. Control options include both feedback [29] and feedforward (sometimes necessitating system identification) [30] in addition to shaping of the commanded trajectory to ameliorate the deleterious effects of interactions between the control and robot modes that reduce appendage pointing and positioning accuracy. Feedback control is arguably the best understood to adjust robot performance by designing the closed loop system [31]. Well-established, classical techniques include proportional plus integral plus derivative (PID) control [32], emphasizing oscillation design and maintenance of stability following a commanded movement trajectory. A common modern control method used to minimize a performance measure is the linear quadratic regulator [33] ubiquitously applied to linear time-invariant systems, while the presumption for robotic space missions used for refueling and resupply particularly must assume time variance, since fuels are being relocated and hardware may be removed and replaced. A less frequently first-used method is feedforward control, elaborated in [34] to be particularly useful if the movements of the space robot are predictable. Feedforward control is also referred to as open-loop control. The recent resurgence of artificial intelligence has not overlooked space robotics, or medical purposes [35,36]. Robots can be trained by machine learning algorithms to compensate for varying tasks and environments. The internet of things (IoT) is another newly conceived possibility for remote robot control and monitoring [37]. 

The vibration of flexible bodies is strongly driven by the nature of the (impact) excitation or rate of application of external forces. Accordingly, the dynamics of physical contact are important, leading to a field of study known as contact dynamics. Such dynamics are modeled using a so-called Hertz model in [38], which studied gripping a non-cooperative spacecraft focusing on contact compliance control. The study highlighted the key nature of the vibration of flexible spacecraft parts, which could lead to repeated continuous collisions. The high flexibility of the robot arms is a key focus of attention for capturing non-cooperative spacecraft. This study presented simulation experiments indicating that compliance control seems key to successful performance. 

The natural vibrational frequency of the robotic arms is dominated by the arms’ masses and structural stiffness (resistance to motion, either translational or rotational). Tracking control of variable stiffness actuators was studied in [39], highlighting robot arm link motor disturbances and variability in actuator stiffnesses (naturally), which may render tracking control schemes ineffective. A mechanism for learning was proposed to compensate for disturbance uncertainties, leading to a novel framework for designing controls, starting with back-stepping tuning of feedback control, then parameter tuning with finite switching. Validation was offered using computer simulations.

A well-known method called input shaping from the 1990s has recently been hybridized in [40,41], seeking to address vibration residuals in flexible, multi-mode systems. Modal analysis is used to decouple the system by transforming to the reference frame defined by the modal (eigen) vectors. The command is shaped in the diagonalized modal reference frame, where the three contending alternatives were versine (sinusoidal), ramp, or cycloid plus ramped sinusoidal. The methods were compared in simulation experiments which validated high robustness to parameter uncertainty. *The general approach is duplicated in this present manuscript comparing bio-inspired whiplash options.*

The study in [42] establishes the present study’s comparative benchmark offered by classical control methods augmented with signal processing filters, and the prequel’s benchmark space robot is the same U.S. Navy system, with natural frequencies of vibration that are so low as to be excited by even low-bandwidth feedback systems. The utilization of versine shaping proved superior to step commands when the structural filters were designed to achieve system stability margins (classical gain margin and phase margin). The study in [43] is an intermediate sequel investigation on the same Navy space robot system, where so-called systems theory methods of Lev Pontryagin were used to develop an open-loop optimal control to minimize maneuver time with quiescent final conditions. The surprising results indicated a “whiplash”-shaped command (initially in a direction opposite to the commanded terminal state) minimized maneuver time. Results were produced in a commercial, pseudospectral optimization software, and then validation was performed analytically using six necessary conditions of optimization: (1) the Hamiltonian minimization condition; (2) adjoint equations; (3) the terminal transversality condition; (4) the Hamiltonian final value condition; (5) the Hamiltonian evolution equation; and (6) Bellman’s principle. Importantly, the biomimicking “whiplash” shaping failed to validate one of the six necessary conditions motivating continuing research (including this present manuscript as a new sequel). The biomimicry whiplash control [43] behavior (depicted earlier in this manuscript in Figure 1) is evaluated in this present study applied to shaping the commanded input rather than as a feedforward control. 

In addition to high structural flexibility, motor torque limits and joint flexibility are additional considerations emphasized in [44] which presented a model-based autonomous generation method for trajectories assuming base excitation and large time delays for any communications with earth. Computer simulations for verification were presented alongside validation by laboratory experiments on an air-bearing table. 

Bio-inspired techniques were specifically highlighted in [45] including the adhesion mechanism and its locomotion system mimicking geckos, spider-inspired actuators, joints inspired by human knees, particle transport by peristaltic motion, and locus-inspired digging mechanisms. Nearly a decade later, NASA presented [46], introducing all-terrain legged rovers inspired by mountain goats, human-like planetary exploration robots, deep drilling inspired by gophers, artificial muscles as actuators, snake-like robotics for traversing narrow openings and passages. Just a few years later, Ellery offered a tutorial review of bio-inspired approaches to robotic manipulation for space debris salvage [47], highlighting sensorimotor control mimicking the human brain for several strategies including (i) sensorimotor planning, learning and control, (ii) optimal feedback control, (iii) impedance control, (iv) predictive control, and (v) Bayesian inferencing. That same year, Ellery also illustrated how to build a biological machine using engineering materials and methods [48]. Just last year, the study in [49] offered an alternative approach applied to a disparate system relative to the research in the present study presented in this manuscript: A biomimetic approach for control of a seven-degrees-of-freedom robotic arm. Namely, central nervous system-based motor control (neural networks with deep learning) is presented as a more directly competing deterministic alternative with the motor control methods presented by Menezes for underwater robotics [50]. Another aspect of the present study is vibration elimination, while an alternative was included in [51], where through slowing the seed and thereby reducing the negative effects of hitting the ground, many species of nuts have evolved differently by producing a rigid layer of protective shell around their seed.

Operationally, highly flexible space robots need to move around large loads of heavy items, potentially leading to large tracking errors. The study in [52] proposed damping–stiffness control including joint dynamics in a comprehensive model utilizing Luenberger observers for unmeasurable quantities. Damping was treated as a feedforward (plus gain), while the feedback is used to suppress perturbations. Verification in simulations hinted at a potential 98% percent improvement, while laboratory experiments merely validated an 88% improvement. 

The dynamics (mathematical models) were the focus of [53], seeking the proper element number for inclusion in appendage models, where the end effector trajectory was controlled in the feedforward. Sliding mode control including gravity effects was proposed in [54] including nonlinear dynamics typically decomposed into separate flexible and rigid subsystems (as was in the present manuscript’s study), but modelling accuracy strongly drove performance. As the control of highly flexible robotic systems becomes more commonplace, a MATLAB^®^/Simulink^®^ toolbox evolved and was presented in [55], effectively reducing the coding burden of future investigations. 

### 1.3. The Current State of the Research Field and Key References

The study in [38] describes substantial on-going proof-of-concept investigations including space debris removal, life-extension services, on-orbit assembly, and manufacturing, while identifying remaining challenges, particularly simulation of the true six-degrees-of-freedom dynamics of large-scale microgravity operations, especially for robotic systems. *The fourth option, so-called “whiplash shaping”, is the bio-inspired method of shaping the input commanded trajectories for the space robot.*

1. Gain stabilization [56]: Tuning of gain to achieve stability of the rigid-body mode. Advantages: simplicity and based on well-known mathematics. Disadvantages: imprecise and uses effort (or equivalently fuel) wastefully compared to more modern methods.
2. Classical second-order structural filtering [56]: Second-order filters designed for each chosen resonance and anti-resonance, usually of the lowest mode or the lowest two modes to ensure stability. Advantages: aids fuel usage of gain stabilization approaches. Disadvantages: mathematic model must have precision and remain not time varying.
3. Input shaping [40,57,58,59]: Modification of open-control frequency content using time-delayed control application. Advantages: existing proofs of mathematical optimality. Disadvantages: lacks robustness.
4. *Whiplash compensation* [60,61]: Initially commanding maneuver in the opposite direction of desired end-state. Advantages: existing proofs of mathematical optimality. Disadvantages: lacks robustness.
5. Rigid-body, minimum-fuel input trajectory shaping: Apply control analytically derived from constrained control-minimization boundary value problem solutions. Advantages: existing proofs of mathematical optimality. Disadvantages: lacks robustness.
6. Single-frequency trajectory shaping: The fashion commanded trajectory from a single sinusoid chosen to avoid mode frequencies of the flexible robot. Advantages: simplicity. Disadvantages: still need accurate mathematical models to properly pick the single frequency.
7. Flatten the curve to improve stability: Use option #2 to compensate for all structural modes seeking to create a magnitude response curve resembling such a curve for a second-order rigid-body system (primary motivation remains increased system stability). Advantages: aids fuel usage of gain stabilization approaches. Disadvantages: mathematic model must have precision and remain not time varying.
8. Flatten the curve to improve trajectory tracking: This option is like option #7, except choosing parts of modes (resonance or anti-resonance) to minimize trajectory tracking errors (proposed in this manuscript). Advantages: aids fuel usage of gain stabilization approaches. Disadvantages: mathematic model must have precision and remain not time varying.
9. Deterministic artificial intelligence: Use physics to define robot self-awareness, while adapting or learning time-varying physical system parameters (e.g., mass, mass moments, stiffness, and damping). Advantages: proofs of optimality, robustness and simple algorithms. Disadvantages: relatively unknown compared to peer methods.
  9.1 Self-awareness statements [62]: Use governing equations from physics to exclusively define robot self-awareness, while prescribing necessary trajectories to be tracked (currently only sinusoidal trajectories and control-minimizing trajectories are in the literature).
  9.2 Adaption or optimal learning [63]: Use classical control methods (e.g., the “M.I.T. Rule” [64] to adapt system parameters to minimize tracking errors or alternatively use least squares estimation methods (e.g., batch, recursive, and extended).

### 1.4. Controversial and Diverging Hypotheses—Literature Gaps

Two disparate paradigms are evident regarding response magnitude curve shaping: flattening the curve to improve stability [42] versus flattening the curve to improve trajectory tracking (to be addressed in this manuscript).

### 1.5. Main Aim of the Work and Highlighting of Principal Conclusions

The goal is to provide the readership an extensive study comparing performances of available options based on multiple figures of merit: necessary fuel expenditure, mean tracking errors, and tracking error deviations.

### 1.6. Novelties Presented

1. Commanded trajectory-shaping options are compared using control effort and tracking accuracy, and recommendations are offered.
2. Feedforward controls are compared using control effort and tracking accuracy, and recommendations are offered.
3. Commanded trajectories are compared with filtered feedback and no feedforward using least control effort tracking accuracy, and recommendations are offered.
4. Mode 1 filtering options are compared using control effort tracking accuracy, and recommendations are offered.
5. Mode 3 filtering options are compared using control effort tracking accuracy, and recommendations are offered.
6. Mode 4 filtering options are compared using control effort tracking accuracy, and recommendations are offered.
7. Overall recommendations are made for selection of commanded trajectories, feedforward controls, and filtered versus unfiltered feedback.
8. The least control effort was achieved with step trajectories, rigid-body optimal feedforward control and unfiltered feedback, while recommendations are offered based on tracking accuracy and control effort.

## 2. Materials and Methods

**Figure 6:**
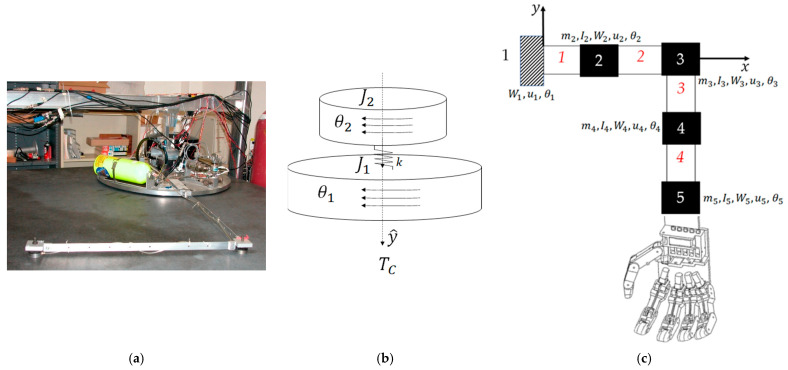
(**a**) Laboratory flexible spacecraft robotic arm (very lightweight) attached to a free-floating planar air-bearing rotational hub. Image used in compliance with image use policy [2], “U.S. Department of Defense photographs and imagery, unless otherwise noted, are in the public domain”. (**b**) Schematic of center-lined, cylindrical, rigid spacecraft. (**c**) The flexible arm is modeled using the lumped-mass technique, where arm mass is distributed to discretized nodes. Images taken from [60] in compliance with respective image use policies [65].

Section 2.1 introduces modeling of the highly flexible space robot using as a benchmark the free-floating (on an air-bearing) flexible spacecraft simulator at the Naval Postgraduate School depicted in Figure 6, which also depicts system diagrams including both rigid-body and flexible robotic appendages. Development of system models (equations) follows next, where the resulting detailed equations are provided in Appendix A. Section 2.2 introduces competing control methodologies and depicts simulation topologies, where detailed simulation codes are provided in Appendix B. 

*Selectable options include commanded trajectories, feedforward controls, feedback controls, and structural filtering.*

### 2.1. Space Robot Modeling

The highly flexible space robot laboratory hardware depicted in Figure 6, subfigure (a) is decomposed into a rigid-body base (simulating the near-rigid spacecraft) depicted in subfigure (b) with highly flexible, lightweight robotic appendages modeled using the finite element method in subfigure (c). The first node of the finite element representation is attached to the spacecraft, while flexible elements (mass and stiffnesses) are lumped at subsequent nodes, where Table 2 conveniently defines variables and nomenclature.

**Table 2:** Table of proximal variables and nomenclature ^1^.

Variable/Acronym	Definition	Variable/Acronym	Definition
$\hat{y}$	Centerline unit vector	k	Appendage stiffness
$T_{C}$	Control torque	$m_{i},I_{i}\forall i=2\ldots 5$	Flexible masses and inertias
$J_{1}$	Main body inertia mass moment	$J_{2}$	Flexible inertia mass moment
$\theta_{1}$	Main body rotation angle	$\theta_{i},W_{i}\forall i=2\ldots 5$	Translation and rotation angles

^1^ Such tables are offered throughout the manuscript to aid readability.

Equations of motion may be derived using at least several disparate methods commonly understood in kinetics: Hamilton’s Principle, Lagrange’s equations, and Chasle’s theorem combining Newton’s Law and Euler’s equations. Since prequel [43] elaborated Hamilton’s Principle, while prequel [42] elaborated Lagrange’s equations, this present manuscript elaborates Chasle’s Theorem. Newton’s Law for translational motion is expressed in Equation (1), where displacements are expressed in coordinates of an inertial reference frame, while similarly Euler’s moment equations are displayed in Equation (2). Expressing motion in coordinates of a non-inertial reference frame modifies Equations (1) and (2) to Equations (3) and (4), respectively. A representative two-node system of equations including internal flexible “spring forces” that resist motion is displayed in Equations (5) and (6), respectively, for each node, and then assembled into matrix-vector notation in Equation (7), where Table 2 conveniently defines variables and nomenclature.

(1) $${\left.F\right|}_{inertial}={\left.ma\right|}_{inertial}={\left.m\dot{v}\right|}_{inertial}={\left.m\ddot{x}\right|}_{inertial}$$

(2) $${\left.T\right|}_{inertial}={\left.J\alpha \right|}_{inertial}={\left.m\dot{\omega }\right|}_{inertial}$$

(3) $$F=m\ddot{x}+m\frac{d\omega }{dt}\times r^{\prime }+2m\omega \times v^{\prime }+m\omega \times \left(\omega \times r^{\prime }\right)$$

(4) $$T=J\dot{\omega }+\omega \times J\omega$$

(5) $$m_{1}{\ddot{x}}_{1}=-k_{1}x_{1}+k_{2}\left(x_{2}-x_{1}\right)$$

(6) $$m_{2}{\ddot{x}}_{2}=-k_{2}\left(x_{2}-x_{1}\right)+F$$

(7) $$\underset{\left[M\right]}{\underbrace{\left[\begin{array}{cc} m_{1} & 0\\ 0 & m_{2} \end{array}\right]}}\underset{\left\{\ddot{x}\right\}}{\underbrace{\left\{\begin{array}{c} {\ddot{x}}_{1}\\ {\ddot{x}}_{2} \end{array}\right\}}}+\underset{\left[K\right]}{\underbrace{\left[\begin{array}{cc} k_{1}+k_{2} & -k_{2}\\ -k_{2} & k_{2} \end{array}\right]}}\underset{\left\{x\right\}}{\underbrace{\left\{\begin{array}{c} x_{1}\\ x_{2} \end{array}\right\}}}=\underset{\left\{F\right\}}{\underbrace{\left\{\begin{array}{c} 0\\ 1 \end{array}\right\}F}}$$

**Table 3:** Table of proximal variables and nomenclature ^1^.

Variable/Acronym	Definition
${\left.F\right|}_{inertial}$ , ${\left.T\right|}_{inertial}$	Externally applied force and torque expressed in inertial coordinates
F , T	Externally applied force and torque expressed in non-inertial coordinates
m , J	Body’s mass and mass moment of inertia
${\left.a\right|}_{inertial}={\left.\dot{v}\right|}_{inertial}={\left.\ddot{x}\right|}_{inertial}$	Resulting accelerations expressed in inertial coordinates
ω , $\dot{\omega }$	Angular velocity and acceleration vectors
$x_{1},x_{2}$ ; ${\ddot{x}}_{1},{\ddot{x}}_{2}$	Translational velocity and acceleration vectors
$k_{1},k_{2}$	Flexible member stiffnesses
$\left[M\right],\left[K\right]$	Assembled matrices of masses and stiffnesses

^1^ Such tables are offered throughout the manuscript to aid readability.

Euler’s moment equations in Equation (2) are elaborated for the flexible space robot in Equation (8) and resembled in Equation (9) to more closely resemble the basic expression of Newton’s Law, where variables and nomenclature are conveniently defined in Table 3.

(8) $$I_{zz}\ddot{\theta }+\sum_{i=1}^{n}D_{i}{\ddot{q}}_{i}+{I_{w}\ddot{\theta }}_{w}=T_{D}$$

(9) $$I_{zz}\ddot{\theta }+\sum_{i=1}^{n}D_{i}{\ddot{q}}_{i}=\sum T$$

**Table 4:** Table of proximal variables and nomenclature ^1^.

Variable/Acronym	Definition
$I_{zz}$	Body principal moment of inertia with respect to *Z*-axis
$\ddot{\theta }$	Angular acceleration of the system rotation angle, θ
D	Rigid–elastic coupling term
$\ddot{q}$	Acceleration in generalized displacement coordinates
$I_{w}$	Reaction wheel principal moment of inertia with respect to *C*, *Z* axis
${\ddot{\theta }}_{W}$	Angular acceleration of the reaction wheel rotation angle, $\theta_{W}$
T	Control torque of the spacecraft reaction wheel
$T_{D}$	Disturbance torques

^1^ Such tables are offered throughout the manuscript to aid readability.

Isolating the first term of Equation (9) leads to Equation (10), and slight arithmetic leads to Equation (11).

(10) $$\ddot{\theta }+\frac{\sum_{i=1}^{n}D_{i}}{I_{zz}}{\ddot{q}}_{i}=\frac{\sum T}{I_{zz}}$$

(11) $$\ddot{\theta }=\frac{\sum T}{I_{zz}}-\frac{\sum_{i=1}^{n}D_{i}}{I_{zz}}{\ddot{q}}_{i}$$

Detailed implementation on the flexible space robot depicted in Figure 6 is included in the appendix to aid repeatability. Mode shapes and (constant) natural vibrational frequencies are obtained by spectral decomposition (i.e., the eigenvalue problem). Meanwhile, mass and moments of inertia (locations of mass) vary, leading to time-invariant frequenices and shapes, thus motivating the proposals in this manuscript with the anticipation of future research into deterministic artificial intelligence. 

### 2.2. Competing Control Design Methodologies

Many options are available in the literature to the robot designer to control the highly flexible system in space. The immediate prequels elaborated gain stabilization, classical second-order structural filtering, input shaping, and whiplash compensation (where whiplash trajectory shaping is implemented in this study). Meanwhile, this present manuscript iterates several remaining trajectory-shaping options: rigid-body minimum-fuel trajectory shaping, single-frequency sinusoid, and options for flattening the magnitude response plot, while establishing some groundwork to prepare for future efforts with deterministic artificial intelligence.

1. Gain stabilization,
2. Classical second-order structural filtering,
3. Input shaping,
4. Whiplash compensation,
5. Rigid-body minimum-fuel input trajectory shaping,
6. Single-frequency trajectory shaping,
7. Flatten the curve to improve stability,
8. Flatten the curve to improve trajectory tracking,
9. Deterministic artificial intelligence:
  9.1 Self-awareness statements, and
  9.2 Adaption or optimal learning.

### 2.3. Selectable Options: Trajectories, Feedforward, Feedback, and Filtering

**Figure 7:**
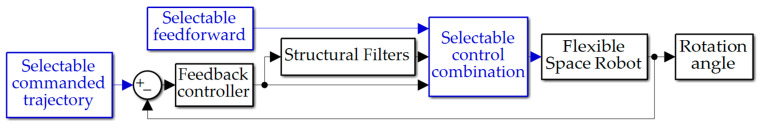
Topology of simulation created in Simulink^®^.

Figure 7 depicts simulations created in SIMULINK^®^ including subsystems for the selectable commanded trajectory, selectable feedforward controls, feedback controller, structural filters, and the selection subsystem to activate feedforward, feedback, and structural filtering. Those subsystems are fed to control the flexible space robot’s subsystem in a unit-feedback loop resulting in a displayable rotation angle. 

#### 2.3.1. Commanded Trajectories

**Figure 8:**
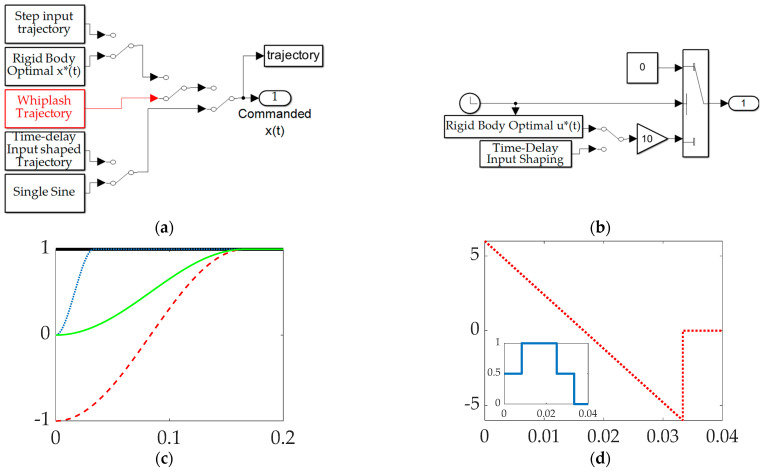
Topology of simulation created in Simulink^®^: (**a**) selectable command trajectory; (**b**) selectable feedforward; (**c**) selectable commanded trajectories: the thick solid black line is the unit step function, the rigid-body minimum fuel is the thin solid green line, ***bio–inspired whiplash trajectory*** is the red dashed line, and the single sinusoidal commanded trajectory is the dotted blue line. (**d**) Feedforward controls: minimum fuel optimal feedforward is the dashed red line, while time-delay input-shaped feedforward is the solid blue line in the inset plot.

The Simulink^®^ subsystem used to select between commanded trajectories is displayed in Figure 8a including ubiquitous step commands, rigid-body control-minimizing optimal commands, whiplash compensation, time-delay input-shaped trajectories, and single sinusoidal commanded trajectories. Figure 8b displays a subsystem used to formulate trajectories that are non-zero only when maneuvering. Meanwhile, Figure 8c,d display notion subsystem outputs. 

#### 2.3.2. Feedback Filtering

**Figure 9:**
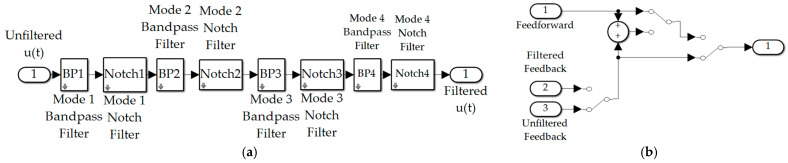
Topology of simulation created in Simulink^®^: (**a**) structural filters; (**b**) selectable control combination.

**Figure 10:**
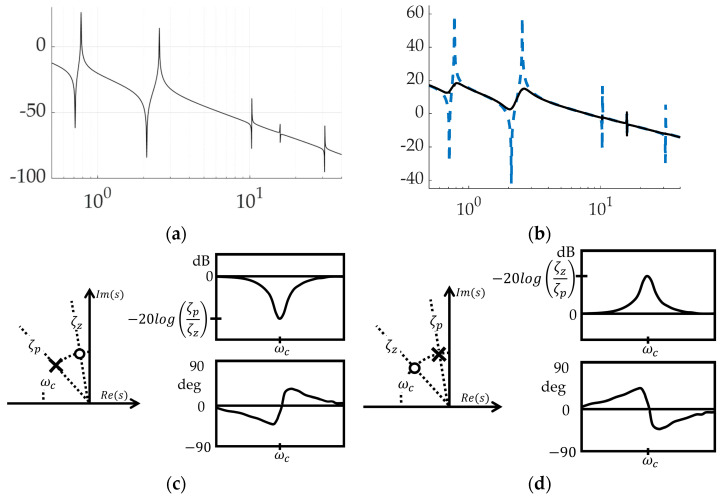
Feedback filtering for structural resonances and anti-resonances: (**a**) frequency response plot of unfiltered, PID controlled space robot with decibel frequency on the abscissa and response magnitude on the ordinant; (**b**) flattened curve, frequency response plot when all four modes are filtered with both bandpass and notch filters with decibel frequency on the abscissa and response magnitude on the ordinant; (**c**) second-order notch filters optionally applied at resonances with decibel frequency on the abscissa and response magnitude on the ordinant of the right-hand subplot with Real (Re) and Imaginary (Im) parts displayed in the left-hand plot; (**d**) second-order bandpass filters optionally applied to anti-resonances with decibel frequency on the abscissa and response magnitude on the ordinant of the right-hand subplot with Real (Re) and Imaginary (Im) parts displayed in the left-hand plot.

Filtering feedback controls is simulated as depicted in Figure 9a, where the iteration of controls is achieved by a subsystem of manual switches depicted in Figure 9b. Activation of all structural filtering (four bandpass filters placed at the spectral location of four anti-resonances plus four notch filters placed at the spectral location of four resonance frequencies) results in modifying the frequency response magnitude plot depicted in Figure 10a,b, where the spikes and dips of resonance and anti-resonance, respectively, have been smoothed by the structural filters. Former analysis designed these filters primarily using stability as motivation, while this present research evaluates several figures of merit including trajectory tracking and control usage. Figure 10c,d, respectively, depict notion impacts of notch and bandpass filters on the frequency response magnitude plot (i.e., Bode plot’s magnitude), where Table 4 provides convenient definitions of proximal variables and nomenclature.

**Table 5:** Table of proximal variables and nomenclature ^1^.

Variable/ Acronym	Definition
Im(s)	Imaginary component of transient response
Re(s)	Real component of transient response
$\zeta_{p}$	Damping ratio of pole in denominator of Equation (12)
$\zeta_{z}$	Damping ratio of zero in numerator of Equation (12)
$\omega_{c}$	Center frequency of filter placement
$\omega_{p}$	Center frequency of filter pole placement in denominator of Equation (12)
$\omega_{z}$	Center frequency of filter zero placement in numerator of Equation (12)
dB	Decibels
log	Base-10 logarithm
Output(s)	Displacement or rotation expressed in Laplace domain
$Input\left(s\right)$	Control force or torque expressed in Laplace domain
$K_{\infty }$	Steady state gain
$\phi_{max}$	Maximum phase lead occurring at frequencies determined by $\zeta_{z}$ and $\zeta_{p}$
$K_{max}$	Maximum gain occurring when $\omega_{p}=\omega_{p}$

^1^ Such tables are offered throughout the manuscript to aid readability.

(12) $$\frac{Output(s)}{Input(s)}=\frac{\frac{s^{2}}{\omega_{z}^{2}}+\frac{2\zeta_{z}}{\omega_{z}^{2}}s+1}{\frac{s^{2}}{\omega_{p}^{2}}+\frac{2\zeta_{p}}{\omega_{p}^{2}}s+1}$$

(13) $$K_{\infty }=40{log}_{10}\left(\omega_{p}/\omega_{z}\right)$$

(14) $$\phi_{max}={cos}^{-1}\left[\frac{{\left(2\zeta_{c}\sqrt{\omega_{p}/\omega_{z}}\right)}^{2}-{\left(\omega_{p}/\omega_{z}-1\right)}^{2}}{{\left(2\zeta_{c}\sqrt{\omega_{p}/\omega_{z}}\right)}^{2}+{\left(\omega_{p}/\omega_{z}-1\right)}^{2}}\right]$$

(15) $$K_{max}=20{log}_{10}\left(\zeta_{z}/\zeta_{p}\right)\;dB$$

(16) $$\omega_{1}/\omega_{c}=\sqrt{2\zeta_{z}\zeta_{p}+1-\sqrt{{\left(2\zeta_{z}\zeta_{p}+1\right)}^{2}-1}}$$

(17) $$\omega_{2}/\omega_{c}=\sqrt{2\zeta_{z}\zeta_{p}+1+\sqrt{{\left(2\zeta_{z}\zeta_{p}+1\right)}^{2}-1}}$$

#### 2.3.3. Feedforward Controls

Feedforward controls are strictly taken as the self-awareness statements in deterministic artificial intelligence, where the intension is to use this manuscript as benchmarks for the seminal development of deterministic artificial intelligence for flexible space robotics in the sequel. For example, presently Equation (8) is modified to Equation (18) by prescribing the motion states (using the chosen commanded trajectories) and using time-invariant estimates of the physical parameters (e.g., mass, mass moments, stiffnesses, and eventually damping). The sequel will modify Equation (9) is modified to Equation (19) by prescribing the motion states (using the chosen commanded trajectories) and using time-invariant estimates of the physical parameters (e.g., mass, mass moments, stiffnesses, and eventually damping). develop adaption and learning methods for the estimates making them time varying. Follow-on work will combine the transport theorem in Equation (18) with the rigid–elastic coupled system in Equation (19) which should permit deterministic artificial intelligence to learn time-varying natural frequencies stemming from time-varying mass, mass moments, stiffnesses, and damping.

(18) $$T\equiv \hat{J}{\dot{\omega }}_{d}+\omega_{d}\times \hat{J}\omega_{d}$$

(19) $${\hat{I}}_{zz}{\ddot{\theta }}_{d}+\sum_{i=1}^{n}{\hat{D}}_{i}{\left({\ddot{q}}_{i}\right)}_{d}\equiv \sum T$$

Selectable options for designing commanded trajectories, feedforward and feedback controls, and structural filtering were elaborated in Section 2.3. Presently concluding the Methods and Materials, Section 3 next presents the parameters used in the simulation experiments, and then presents the results of many experiments. 

## 3. Results

This section provides a concise and precise description of the experimental results favoring multi-plots with accompanying tables of comparative figures of merit to aid the readership’s efforts ascertaining the relative efficacy of the approaches presented. Presented next is the data interpretation, as well as the experimental conclusions that can be drawn, culminating in a very large table of thirteen of the best approaches (of the twenty-six approaches iterated), displayed with comparative figures of merit. Mini summaries are provided, allowing the reader to discard thoughts of relatively inferior methods, while continuing to the next set of comparisons, eventually narrowing to a grouping of the best available options of those surveyed. The simulation parameters are provided in Table 5 to aid repeatability of the presented results. 

### 3.1. Comparing Commanded Trajectories with Unfiltered Feedback

**Figure 11:**
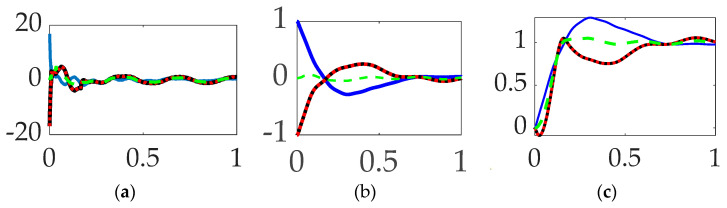
Trajectory command comparative simulation experiments performed in Simulink^®^ with normalized time on the abscissae. The solid blue line indicates step trajectory, no feed-forward, unfiltered; the solid black line indicates whiplash trajectory, no feedforward, unfiltered; the red dotted line indicates time-delayed input-shaped trajectory, no feedforward, unfiltered, where the ordinants display: (**a**) control in [Newton meters], (**b**) tracking error in [degrees], and (**c**) rotation angle in [degrees]. Qualitative results correspond to quantitative figures of merit in Table 6. Rigid-body minimum-fuel input trajectory shaping performed so poorly as to not be presentable.

The first options experiments compared disparate options for comparing commanded trajectories with unfiltered, classical PID feedback control. Space robot rotation angles are displayed versus time (scaled to unity) in Figure 11 with corresponding figures of merit in Table 6 revealing obviously superior tracking performance of using single-sinusoidally shaped commanded trajectories without feedforward control and unfiltered classical PID feedback control. Orders-of-magnitude improvements in trajectory tracking mean error and deviations is achievable with more than three-fold increase in fuel utilization (i.e., control effort). 

**Interim summary.** *When comparing commanded trajectories, step trajectories surprisingly led to the least control effort, while single-sinusoid trajectories produce the most accurate tracking, with 150% more control effort. **Bio-inspired whiplash compensation performed essentially as well as time-delayed input shaping.***

### 3.2. Comparing Feedforward Controls with Unfiltered Feedback

**Figure 12:**
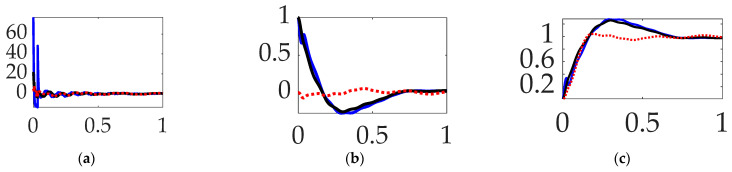
Selectable feedforward comparative simulation experiments performed in Simulink^®^ with normalized time on the abscissae. The solid blue line indicates step trajectory, rigid-body optimal feed-forward, unfiltered; the solid black line indicates step trajectory, time-delay input shaping feedforward, unfiltered; the red dotted line indicates single-sine trajectory, time-delay input shaping feedforward, unfiltered, where the ordinants display: (**a**) control in [Newton meters], (**b**) tracking error in [degrees], and (**c**) rotation angle in [degrees]. Qualitative results correspond to quantitative figures of merit in Table 7. Single-sinusoid trajectory, rigid-body optimal feedforward, unfiltered performed so poorly as to not be presentable.

Having compared trajectory-shaping options in Section 3.1, this section includes the results of direct comparison of disparate options for feedforward control in the presence of unfiltered, classical PID feedback control. Figure 12 reveals that relatively superlative performance is obtained by using single-sinusoidally shaped, commanded trajectories with time-delay input-shaped feedforward control in the presence of unfiltered classical feedback controls, where figures of merit in Table 7 reveal the increased trajectory tracking performance necessitates a non-trivial increase in fuel expenditure (control effort).

**Interim summary.** When comparing feedforward controls, rigid-body optimal feedforward with step trajectory command surprisingly led to the least control effort, while time-delay input-shaped feedforward with single-sinusoid trajectories commanded produced the most accurate tracking, with 280% more control effort.

### 3.3. Comparing Commanded Trajectories with Filtered Feedback

**Figure 13:**
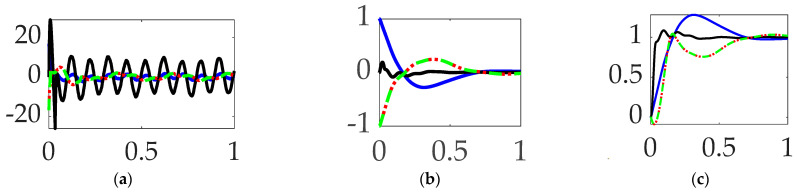
Selectable commanded trajectory comparative simulation experiments performed in Simulink^®^ with normalized time on the abscissae. The solid blue line indicates step trajectory, no feedforward, filtered feedback; the solid black line indicates rigid-body optimal trajectory, no feedforward, filtered feedback; the red dotted line indicates whiplash trajectory, no feedforward, filtered feedback; and the green dashed line indicates single-sine trajectory, no feedforward, filtered feedback, where the ordinants display: (**a**) control in [Newton meters], (**b**) tracking error in [degrees], and (**c**) rotation angle in [degrees]. Qualitative results correspond to quantitative figures of merit in Table 8.

Having discerned the advantages of single-sinusoidally shaped, commanded trajectories and time-delay input-shaped feedforward control, this section repeats the comparison in Section 3.1 (which included only unfiltered feedback), but this time iterates the options when structural filters are use. Figure 13 reveals the lowest control effort (i.e., fuel usage) with nominal tracking performance using unshaped step commands, no feedforward controls with structurally filtered feedback; however, substantially improved target tracking performance is achievable at using higher control efforts by commanding optimal (fuel-minimizing) trajectories that are constrained by rigid-body dynamics equations. 

**Interim summary.** When comparing commanded trajectories with filtered feedback and no feedforward, step trajectory commands surprisingly led to the least control effort, while rigid-body optimal trajectories achieved an order of magnitude higher accuracy with 2345% more control effort.

### 3.4. Comparing Mode 1 Filtering with Single-Sinusoidal Trajectories and No Feedforward

**Figure 14:**
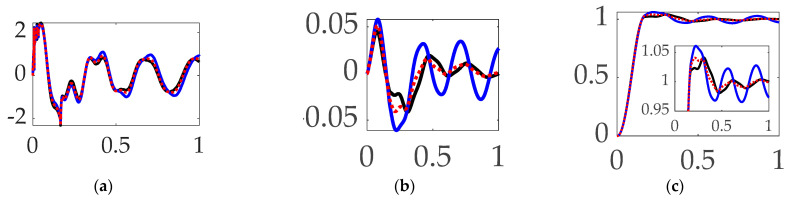
Selectable mode 1 feedback filtering comparative simulation experiments performed in Simulink^®^ with normalized time on the abscissae. The solid black line indicates single-sine trajectory, no feedforward, mode 1 bandpass filtered; the solid blue line indicates single-sine trajectory, no feedforward, mode 1 notch filtered; the red dotted line indicates single-sine trajectory, no feedforward, mode 1 bandpass and notch filtered, where the ordinants display: (**a**) control in [Newton meters], (**b**) tracking error in [degrees], and (**c**) rotation angle in [degrees]. Qualitative results correspond to quantitative figures of merit in Table 8.

The final sections of the manuscript present experiments with iterated structural filtering: mode 1, mode 2, mode 3, mode 4, and then all of modes 1–4. Single-sinusoidally commanded trajectories are carried over from Section 3.1, Section 3.2 and Section 3.3, identified as a burgeoning best practice. This section iterates compensation of the lowest (first) flexible mode by sequentially compensating for the anti-resonance, the resonance, and then both modal features. The prequel literature predominantly designs structural filters for the sake of stability, often leading to emphasizing notch filtering the first resonant peak, while the results here (designed for target tracking performance rather than stability) indicate superior performance is obtainable by only compensating for the first anti-resonance frequency. This assertion is buttressed by the qualitative results in Figure 14, the quantitative companion of which is displayed in Table 9. Selectable mode 1 feedback filtering comparative simulation experiments performed in Simulink^®^. Quantitative figures of merit correspond to qualitative results in Figure 14′s display of meaningful performance figures of merit. 

**Interim summary.** *When comparing mode 1 filtering options bandpass only not surprisingly led to the least control effort, but surprisingly also produced the most accurate tracking.*

### 3.5. Comparing Mode 2 Filtering with Single-Sinusoidal Trajectories and No Feedforward

**Figure 15:**
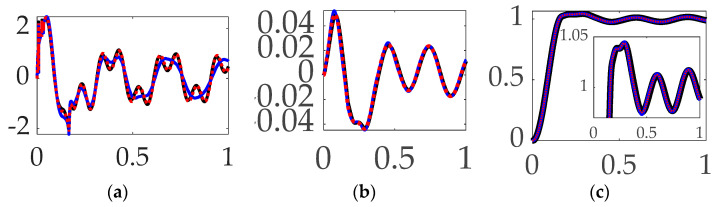
Selectable mode 2 feedback filtering comparative simulation experiments performed in Simulink^®^ with normalized time on the abscissae. The solid black line indicates single-sine trajectory, no feedforward, mode 2 bandpass filtered; the solid blue line indicates single-sine trajectory, no feedforward, mode 2 notch filtered; the red dotted line indicates single-sine trajectory, no feedforward, mode 2 bandpass and notch filtered, where the ordinants display: (**a**) control in [Newton meters], (**b**) tracking error in [degrees], and (**c**) rotation angle in [degrees]. Qualitative results correspond to quantitative figures of merit in Table 10.

Similar to Section 3.4′s comparison of the compensation of the first mode, this section sequentially compensates for the second mode’s anti-resonance, resonance, and then both modal components. The results are displayed in Figure 15 which reveal marginal qualitative differences that are validated by quantitative results in Table 10.

**Interim summary.** When comparing mode 2 filtering options bandpass and notch led to the least control effort, but surprisingly accurate tracking results were inconsistent.

### 3.6. Comparing Mode 3 Filtering with Single-Sinusoidal Trajectories and No Feedforward

**Figure 16:**
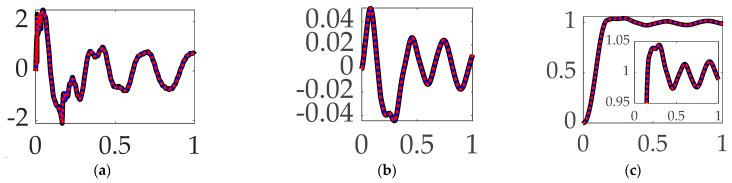
Selectable mode 3 feedback filtering comparative simulation experiments performed in Simulink^®^ with normalized time on the abscissae. The solid black line indicates single-sine trajectory, no feedforward, mode 3 bandpass filtered; the solid blue line indicates single-sine trajectory, no feedforward, mode 3 notch filtered; the red dotted line indicates single-sine trajectory, no feedforward, mode 3 bandpass and notch filtered, where the ordinants display: (**a**) control in [Newton meters], (**b**) tracking error in [degrees], and (**c**) rotation angle in [degrees]. Qualitative results correspond to quantitative figures of merit in Table 10.

Continuing the evaluation to the third structural mode, like Section 3.4 and Section 3.5, this paragraph presents the experiment results of sequentially compensating for the third mode’s anti-resonance, resonance, and then both modal components. Similar to the results achieved compensating for the first resonant mode, compensation of the third mode’s bandpass alone proved superior. In this instance, the results are clearly marginal, evidenced in the qualitative displays of Figure 16, where the small differences are quantitatively displayed in Table 11.

**Interim summary.** When comparing mode 3 filtering options bandpass led to the least control effort and also produced the most accurate tracking accuracy.

### 3.7. Comparing Mode 4 Filtering with Single-Sinusoidal Trajectories and No Feedforward

**Figure 17:**
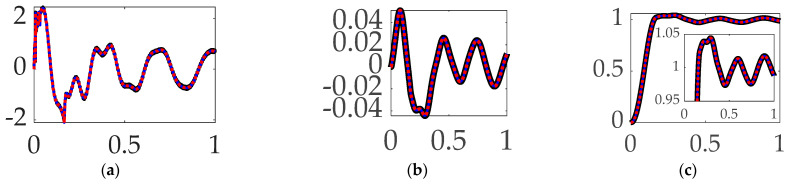
Selectable mode 4 feedback filtering comparative simulation experiments performed in Simulink^®^ with normalized time on the abscissae. The solid black line indicates single-sine trajectory, no feedforward, mode 4 bandpass filtered; the solid blue line indicates single-sine trajectory, no feedforward, mode 4 notch filtered; the red dotted line indicates single-sine trajectory, no feedforward, mode 4 bandpass and notch filtered, where the ordinants display: (**a**) control in [Newton meters], (**b**) tracking error in [degrees], and (**c**) rotation angle in [degrees]. Qualitative results correspond to quantitative figures of merit in Table 12.

The three immediately preceding sections of this manuscript iteratively investigated the first three structural modes, while this paragraph iteratively compensates for the modal components of the fourth structural mode: the anti-resonance, resonance, and then both. Figure 17 (like Figure 16′s qualitative results) reveal marginal results, where inspection of the quantitative results in Table 12 indicate the repeated trend: compensation for the anti-resonance has the largest impact on target tracking errors. 

**Interim summary.** When comparing mode 4 filtering options bandpass led to the least control effort and also produced the most accurate tracking accuracy.

### 3.8. Comparing Modes 1–4 Filtering with Single-Sinusoidal Trajectories and No Feedforward

**Figure 18:**
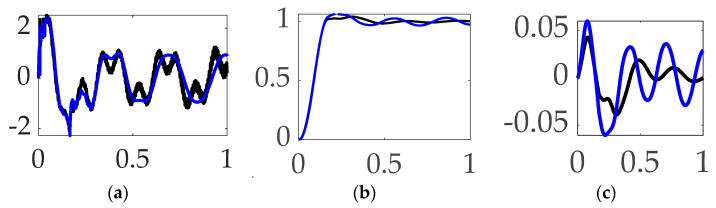
Selectable modes 1–4 feedback filtering comparative simulation experiments performed in Simulink^®^ with normalized time on the abscissae. The solid black line indicates single-sine trajectory, no feedforward, modes 1–4 bandpass filtered; the solid blue line indicates single-sine trajectory, no feedforward, modes 1–4 notch filtered, where the ordinants display: (**a**) control in [Newton meters], (**b**) tracking error in [degrees], and (**c**) rotation angle in [degrees]. Qualitative results correspond to quantitative figures of merit in Table 13.

Section 3.1, Section 3.2, Section 3.3, Section 3.4, Section 3.5, Section 3.6 and Section 3.7 iteratively examined compensation of individual modal components, while this section simultaneously compensates for all four modes’ anti-resonances followed by all round modes’ resonances. Definite differences are immediately apparent in Figure 18, where the quantitative figures of merit in Table 13 re-validate the discoveries of Section 3.1, Section 3.2, Section 3.3, Section 3.4, Section 3.5, Section 3.6 and Section 3.7: compensation for the anti-resonance alone has the biggest impact on target tracking performance (with a reminder: the opposite result comes from compensating for stability rather than tracking performance). 

**Interim summary.** When comparing modes 1–4 filtering options bandpass not only led to the least control effort but also produced the most accurate tracking accuracy.

### 3.9. Comparison of the Best Options Studies

This paragraph assembles and compares thirteen common options for controlling highly flexible space robotics providing advice to the readership: should filtered or unfiltered feedback be used? Should feedforward techniques be considered? Is command trajectory shaping effective? From the experiments presented, interesting mixtures of options illustrate efficacy. Table 14 contains a summary of figures of merit achieved by thirteen disparate combinations of available options: input shaping, feedforward and/or feedback control and optional structural filtering. 

**Interim summary.** The least control effort was achieved with step trajectories, rigid-body optimal feedforward control and unfiltered feedback, and the effort was 75% less than the average. The best mean tracking error was achieved with sinusoidal trajectories, no feedforward, mode 2 notch filtered, and the tracking error mean was 98% better than the average, while the control effort was 330% higher than the minimum available option. The best tracking error deviation was achieved with sinusoidal trajectories, no feedforward, mode 1–4 bandpass filtered, and the tracking error deviation was 80% better than the average, while the control effort was 42% higher than the minimum available option.

## 4. Discussion

**Figure 19:**
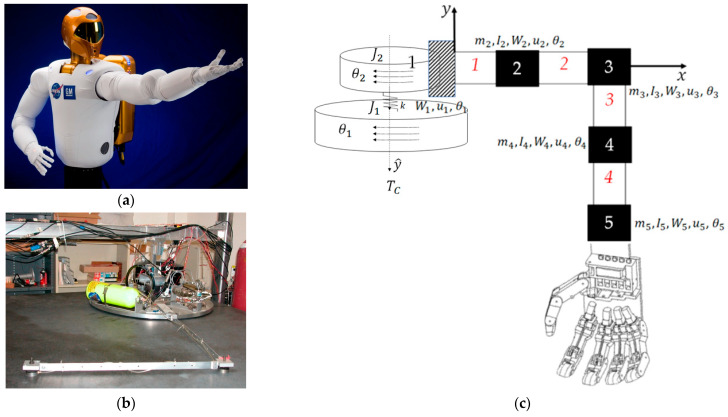
Space robots with cylindrical center rigid bodies and highly flexible appendages. (**a**) NASA’s first humanoid space robot. Image credit: NASA [3]. (**b**) Laboratory flexible rotational spacecraft hub with a free-floating, planar air-bearing, very light robotic arm, the schematic of which is displayed in subfigure (**c**).

Pictures of the machine design are replicated in Figure 19 as a late reminder. Next, this section discusses the maneuver results and how they can be interpreted from the perspective of previous studies and of the working hypotheses. The findings and their implications are discussed in the broadest context possible. Future research directions are also highlighted. 

The top seven revelations follow:

1. Bio-inspired trajectory shaping (modified from a time-minimization control per [43]) seems confounded in the presence of classical, unfiltered feedback. While the technique performed well, it was not the exemplary option when compared to the multitude of other available options examined.
2. When comparing commanded trajectories, step trajectories surprisingly led to the least control effort, while single-sinusoid trajectories produce the most accurate tracking, with 150% more control effort. The bio-inspired whiplash shaping was optimized in the cited literature for minimum time in a feedforward control sense, while this sequel reveals that the solution is not minimum effort (fuel), nor minimum time in the presence of feedback.
3. When comparing feedforward controls, rigid-body optimal feedforward with step trajectory command surprisingly led to the least control effort, while time-delay input-shaped feedforward with single-sinusoid trajectories commanded produced the most accurate tracking, with 280% more control effort.
4. When comparing commanded trajectories with filtered feedback and no feedforward, step trajectory commands surprisingly led to the least control effort, while rigid-body optimal trajectories achieved an order of magnitude higher accuracy with 2345% more control effort.
5. When comparing mode 1 filtering options bandpass alone not surprisingly led to the least control effort, but surprisingly also produced the most accurate tracking.
6. When comparing mode 3 filtering options bandpass led to the least control effort and also produced the most accurate tracking accuracy.
7. When comparing mode 4 filtering options bandpass led to the least control effort and also produced the most accurate tracking accuracy.
8. The least control effort was achieved with step trajectories, rigid-body optimal feedforward control and unfiltered feedback, and the effort was 75% less than the average. The best mean tracking error was achieved with sinusoidal trajectories, no feedforward, mode 2 notch filtered, and the tracking error mean was 98% better than the average, while the control effort was 330% higher than the minimum available option. The best tracking error deviation was achieved with sinusoidal trajectories, no feedforward, mode 1–4 bandpass filtered, and the tracking error deviation was 80% better than the average, while the control effort was 42% higher than the minimum available option.

## 5. Conclusions

The research in [42] studied iterations of classical feedback control augmented with structural filters taken from the discipline of signal processing (e.g., notch filters and bandpass filters), where the options were iterated to maximize classical stability margins (i.e., gain margin and phase margin). Alternatively, the feedback-related sections of this investigation iterated the same options towards a goal of minimizing trajectory tracking error.

Meanwhile, open loop optimal feedforward in [43] was provided for minimizing the maneuver time without the presence of feedback or filtering. The results were bio-inspired, but the time-minimizing results did not rank in the top three performances in the present study seeking the best options for target tracking error. 

### 5.1. Controversial or Unexpected Results

The study in [42] indicates the best selection of options to maximize classical stability margins (i.e., gain margin and phase margin) was attained by only compensating for the first complete flexible mode (resonance and anti-resonance) with notch and bandpass filters, respectively, with step commands shaped by novel single-sinusoidal trajectories. 

Meanwhile, the results presented in the present manuscript indicate split results based on lowest control effort, least tracking error mean, or tracking error deviation. 

#### 5.1.1. Best Control Effort

For the best trajectory tracking measured by least control effort, unfiltered feedback (no notch or bandpass filters) is preferred when unshaped step inputs feed rigid-body optimal feedforward controls, as displayed in Table 15. Thus, unshaped rigid-body optimal feedforward with no filtering in the feedback channel is most appropriate for instances when fuel minimization is strictly required, particularly when traversing long trajectories from initial starting points towards targeted space items (as opposed to close-proximity operations). 

#### 5.1.2. Best Tracking Error Mean

For the best trajectory tracking measured by the lowest mean, unfiltered feedback (no notch or bandpass filters) is the preferred step inputs and rigid-body optimal feedforward controls, as displayed in Table 15. Thus, step inputs with rigid-body optimal feedforward with unfiltered feedback are best for close-proximity operations, especially preparing for grasping operations. 

#### 5.1.3. Best Tracking Error Deviation

For the best trajectory tracking measured by lowest deviation, sinusoidally shaped commanded trajectories with no feedforward but bandpass- only filtering of modes should be used, as displayed in Table 15. During close-proximity operations, grasping necessitates precision pointing and minimal error deviation, and thus sinusoidal-shaped commanded trajectories with no feedforward and bandpass-only mode filtering should be used. 

### 5.2. Recommended Future Reseach

A major motivation of the present study is to evaluate the various efficacies of disparate input shaping techniques available to the recently proposed deterministic artificial intelligence method that necessitates such. 

#### Deterministic Artificial Intelligence

Following the introduction of the feedforward by Cooper/Heidlauf in 2017 [62] applied to chaotic circuits, the seminal introduction of the technique (including both normal optimal feedforward and feedback) was offered by Smeresky/Rizzo [63] applied to spacecraft, despite not yet bearing the name of the technique back in the year 2020. Following the publication of comparative prequels [42,43], the natural sequels are recommended here as future research. Utilize the trajectories evaluated here for spacecraft as inputs to the deterministic artificial intelligence method to formulate a seminal offering applied to highly flexible space robotics. 

## Figures and Tables

**Table 6:** Trajectory command comparative simulation experiments performed in Simulink^®^. Quantitative figures of merit correspond to qualitative results in Figure 11.

Control Methods ^1^	Control Effort	Tracking Error Mean	Tracking Error Deviation
Step trajectory, no feedforward, unfiltered	**0.27662**	0.025967	0.29883
***Bio-inspired whiplash trajectory***, no feedforward, unfiltered	1.0997	–0.026376	0.2936
Time-delayed input-shaped trajectory, no feedforward, unfiltered	1.0997	–0.026376	–0.27936
Single-sinusoid trajectory, no feedforward, unfiltered	0.69228	**0.00052658**	**0.025702**

^1^ Rigid-body minimum-fuel input trajectory shaping performed so poorly as to not be presentable.

**Table 7:** Selectable feedforward comparative simulation experiments performed in Simulink^®^. Quantitative figures of merit correspond to qualitative results in [degrees].

Control Methods ^1^	Control Effort	Tracking Error Mean	Tracking Error Deviation
Step trajectory, rigid-body optimal feedforward, unfiltered	**0.15916**	0.030193	0.29752
Step trajectory, time-delay input-shaped feedforward, unfiltered	0.211539	0.026717	0.27847
Single-sinusoid trajectory, rigid-body optimal feedforward, unfiltered	294.3845	6.1359	3.9368
Single-sinusoid trajectory, time-delay input-shaped feedforward, unfiltered	0.61639	**–0.0014197**	**0.026812**

^1^ Rigid-body minimum-fuel input trajectory shaping performed so poorly as to not be presentable.

**Table 8:** Selectable commanded trajectories with filtered feedback (and no feedforward) comparative simulation experiments performed in Simulink^®^. Quantitative figures of merit correspond to qualitative results in Table 8.

Control Methods ^1^	Control Effort	Tracking Error Mean	Tracking Error Deviation
Step trajectory, no feedforward, filtered feedback	**0.028957**	0.026908	0.30033
Rigid-body optimal trajectory, no feedforward, filtered feedback	3.0251	**0.0020571**	**0.040348**
Bio-inspired whiplash, no feedforward, filtered feedback	0.70804	–0.023355	0.27816
Single-sine trajectory, no feedforward, filtered feedback	0.70804	–0.023355	0.27816

^1^ Rigid-body minimum-fuel input trajectory shaping performed so poorly as to not be presentable.

**Table 9:** Selectable mode 1 feedback filtering comparative simulation experiments performed in Simulink^®^. Quantitative figures of merit correspond to qualitative results in Figure 14.

Single-Sine Trajectory, No Feedforward, Iterated Feedback Filtering	Control Effort	Tracking Error Mean	Tracking Error Deviation
Mode 1 bandpass filtered	**0.62207**	**0.00046139**	**0.018780**
Mode 1 notch filtered	0.89711	0.00046872	0.029906
Mode 1 Bandpass and notch filtered	0.71577	0.00058161	0.020662

**Table 10:** Selectable mode 2 feedback filtering comparative simulation experiments performed in Simulink^®^. Quantitative figures of merit correspond to qualitative results in Figure 15.

Single-Sine Trajectory, No Feedforward, Iterated Feedback Filtering	Control Effort	Tracking Error Mean	Tracking Error Deviation
Mode 2 bandpass filtered	0.47683	0.00026452	**0.022876**
Mode 2 notch filtered	0.68856	**0.00013103**	0.023599
Mode 2 Bandpass and notch filtered	**0.41301**	0.00016559	0.023188

**Table 11:** Selectable mode 3 feedback filtering comparative simulation experiments performed in Simulink^®^. Quantitative figures of merit correspond to qualitative results in Figure 16.

Single-Sine Trajectory, No Feedforward, Iterated Feedback Filtering	Control Effort	Tracking Error Mean	Tracking Error Deviation
Mode 3 bandpass filtered	**0.67211**	**0.00015387**	**0.023116**
Mode 3 notch filtered	0.68804	0.00025072	0.023377
Mode 3 Bandpass and notch filtered	0.68986	0.00017818	0.023274

**Table 12:** Selectable mode 4 feedback filtering comparative simulation experiments performed in Simulink^®^. Quantitative figures of merit correspond to qualitative results in Figure 12.

Single-Sine Trajectory, No Feedforward, Iterated Feedback Filtering	Control Effort	Tracking Error Mean	Tracking Error Deviation
Mode 4 bandpass filtered	**0.65649**	**0.00075748**	**0.023116**
Mode 4 notch filtered	0.69137	0.00330010	0.023697
Mode 4 Bandpass and notch filtered	0.68994	0.00094136	0.023266

**Table 13:** Selectable mode feedback filtering comparative simulation experiments performed in Simulink^®^. Quantitative figures of merit correspond to qualitative results in Figure 13.

Single-Sine Trajectory, No Feedforward, Iterated Feedback Filtering	Control Effort	Tracking Error Mean	Tracking Error Deviation
Modes 1–4 bandpass filtered	**0.22672**	**0.0010466**	**0.017807**
Modes 1–4 notch filtered	**0.90941**	**0.0038787**	**0.030924**

**Table 14:** Comparison of performance figures of merit: effort and tracking errors.

Control Methods ^1^	Control Effort	Tracking Error Mean	Tracking Error Deviation
Bio-inspired whiplash trajectory, no feedforward, unfiltered feedback	1.0997	–0.026376	0.2936
Bio-inspired whiplash, no feedforward, filtered feedback	0.70804	–0.023355	0.27816
Rigid-body optimal trajectory, no feedforward, filtered feedback	3.0251	0.0020571	0.040348
Time-delayed input-shaped trajectory, no feedforward, unfiltered	1.0997	–0.026376	–0.27936
Step trajectory, no feedforward, unfiltered	0.27662	0.025967	0.29883
Step trajectory, no feedforward, filtered feedback	0.028957	0.026908	0.30033
Step trajectory, rigid-body optimal feedforward, unfiltered	**0.15916**	0.030193	0.29752
Single-sinusoid trajectory, no feedforward, unfiltered	0.69228	0.00052658	0.025702
Single-sinusoid trajectory, time-delay input-shaped feedforward, unfiltered	0.61639	–0.0014197	0.026812
Sinusoidal trajectories, no feedforward, mode 1 bandpass filtered	0.62207	0.00046139	0.018780
Sinusoidal trajectories, no feedforward, mode 2 bandpass filtered	0.47683	0.00026452	0.022876
Sinusoidal trajectories, no feedforward, mode 2 notch filtered	0.68856	**0.00013103**	0.023599
Sinusoidal trajectories, no feedforward, mode 2 bandpass and notch filtered	0.41301	0.00016559	0.023188
Sinusoidal trajectories, no feedforward, mode 3 bandpass filtered	0.67211	0.00015387	0.023116
Sinusoidal trajectories, no feedforward, mode 4 bandpass filtered	0.65649	0.00075748	0.023116
Sinusoidal trajectories, no feedforward, mode 1–4 bandpass filtered	0.22672	0.0010466	**0.017807**
**Average**	0.658023	0.007386	0.087848

^1^ Rigid-body minimum-fuel input trajectory shaping performed so poorly as to not be presentable.

**Table 15:** Top three performances in the present study.

Control Methods ^1^	Control Effort	Tracking Error Mean	Tracking Error Deviation
Single-sinusoid trajectory, time-delay input-shaped feedforward, unfiltered	−6%	**−119%**	−69%
Step trajectory, rigid-body optimal feedforward, unfiltered	**−96%**	264%	242%
Sinusoidal trajectories, no feedforward, mode 1–4 bandpass filtered	−66%	−86%	**−80%**

^1^ Rigid-body minimum-fuel input trajectory shaping performed so poorly as to not be presentable.

**Figure A1:**
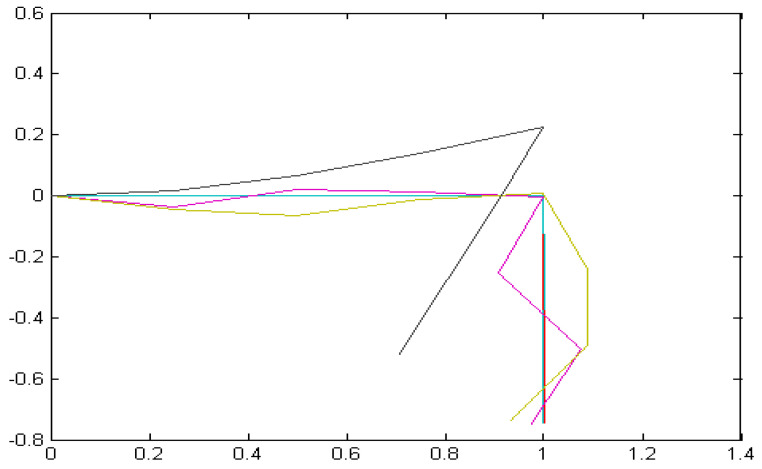
Normalized mode shapes (modal coordinates) displayed in physical coordinates with normalized length on the abscissa in meters and displacements in meters on the ordinant, where each mode shape is annotated by a different color.

## Data Availability

Data supporting reported results can be obtained by contacting the corresponding author.

## Appendix A. Elaboration of Modal System Identification on the Flexible Robot System

Substitute into Equation (19):

(A1)
$$\ddot{\theta }+\frac{\sum_{i=1}^{n}D_{i}\left(-2\xi \omega_{i}I_{zz}\right)}{I_{zz}\left(I_{zz}-D_{i}\sum_{i=1}^{n}D_{i}\right)}{\dot{q}}_{i}+\frac{\sum_{i=1}^{n}D_{i}\left(\omega_{i}^{2}I_{zz}\right)}{I_{zz}\left(I_{zz}-D_{i}\sum_{i=1}^{n}D_{i}\right)}q_{i}+\frac{\sum_{i=1}^{n}D_{i}\left(-D_{i}T\right)}{I_{zz}\left(I_{zz}-D_{i}\sum_{i=1}^{n}D_{i}\right)}=\frac{T}{I_{zz}}$$

(A2)
$$\ddot{\theta }=\frac{2\xi \omega_{i}\sum_{i=1}^{n}D_{i}}{I_{zz}-D_{i}\sum_{i=1}^{n}D_{i}}{\dot{q}}_{i}+\frac{\omega_{i}^{2}\sum_{i=1}^{n}D_{i}}{I_{zz}-D_{i}\sum_{i=1}^{n}D_{i}}q_{i}+\frac{TD_{i}\sum_{i=1}^{n}D_{i}}{I_{zz}\left(I_{zz}-D_{i}\sum_{i=1}^{n}D_{i}\right)}=\frac{T}{I_{zz}}$$

(A3)
$$\ddot{\theta }=\frac{2\xi \omega_{i}\sum_{i=1}^{n}D_{i}}{I_{zz}-D_{i}\sum_{i=1}^{n}D_{i}}{\dot{q}}_{i}+\frac{\omega_{i}^{2}\sum_{i=1}^{n}D_{i}}{I_{zz}-D_{i}\sum_{i=1}^{n}D_{i}}q_{i}+\frac{T\left(D_{i}\sum_{i=1}^{n}D_{i}+I_{zz}-D_{i}\sum_{i=1}^{n}D_{i}\right)}{I_{zz}\left(I_{zz}-D_{i}\sum_{i=1}^{n}D_{i}\right)}$$

(A4)
$$\ddot{\theta }=\frac{2\xi \omega_{i}\sum_{i=1}^{n}D_{i}}{I_{zz}-D_{i}\sum_{i=1}^{n}D_{i}}{\dot{q}}_{i}+\frac{\omega_{i}^{2}\sum_{i=1}^{n}D_{i}}{I_{zz}-D_{i}\sum_{i=1}^{n}D_{i}}q_{i}+\frac{TI_{zz}}{I_{zz}\left(I_{zz}-D_{i}\sum_{i=1}^{n}D_{i}\right)}$$

(A5)
$$\ddot{\theta }=\frac{2\xi \omega_{i}\sum_{i=1}^{n}D_{i}}{I_{zz}-D_{i}\sum_{i=1}^{n}D_{i}}{\dot{q}}_{i}+\frac{\omega_{i}^{2}\sum_{i=1}^{n}D_{i}}{I_{zz}-D_{i}\sum_{i=1}^{n}D_{i}}q_{i}+\frac{T}{\left(I_{zz}-D_{i}\sum_{i=1}^{n}D_{i}\right)}$$

Recall the expressions for the rigid elastic coupling using modal coordinates: $D_{i}=\int_{F}\left(x_{F}\phi_{i}^{y}-y_{F}\phi_{i}^{x}\right)dm$ , where ϕ ’s are mode shapes from finite element analysis using the eigenvalues of K/m (stiffness/mass). The system stiffness matrix is included in Table A1 and the mass matrix in Table A2, resulting in the natural frequencies and mode shapes for the flexible system in Table A3 and Table A4.

**Table A1:** Stiffness matrix [K] ^1^.

	W2	θ2	W3	θ3	W4	θ4	W5	θ5	U6	θ6	u7	θ7	u8	θ8
**W2**	958.8179	0.0000	−479.409	59.9261	0	0	0	0	0	0	0	0	0	0
**θ2**	0.0000	19.9754	−59.9261	4.9938	0	0	0	0	0	0	0	0	0	0
**W3**	−479.409	−59.926	958.8179	0.0000	−479.409	59.9261	0	0	0	0	0	0	0	0
**θ3**	59.9261	4.9938	0.0000	19.9754	−59.9261	4.9938	0	0	0	0	0	0	0	0
**W4**	0	0	−479.409	−59.926	958.8179	0.0000	−479.409	59.9261	0	0	0	0	0	0
**θ4**	0	0	59.9261	4.9938	0.0000	19.9754	−59.9261	4.9938	0	0	0	0	0	0
**W5**	0	0	0	0	−479.409	−59.926	479.409	−59.926	0	0	0	0	0	0
**θ5**	0	0	0	0	59.9261	4.9938	−59.9261	19.9754	−59.9261	4.9938	0	0	0	0
**U6**	0	0	0	0	0	0	0	−59.926	958.8179	0.0000	−479.409	59.9261	0	0
**θ6**	0	0	0	0	0	0	0	4.9938	0.0000	19.9754	−59.9261	4.9938	0	0
**U7**	0	0	0	0	0	0	0	0	−479.409	−59.926	958.8179	0.0000	−479.409	59.9261
**θ7**	0	0	0	0	0	0	0	0	59.9261	4.9938	0.0000	19.9754	−59.9261	4.9938
**U8**	0	0	0	0	0	0	0	0	0	0	−479.409	−59.926	479.4089	−59.926
**θ8**	0	0	0	0	0	0	0	0	0	0	59.9261	4.9938	−59.9261	9.9877

^1^ Notice state sequence alternates translation, then rotation at each node.

**Table A2:** Mass matrix [M] ^1^.

Mass	W2	θ2	W3	θ3	W4	θ4	W5	θ5	U6	θ6	u7	θ7	u8	θ8
**W2**	0.4761	0.0000	0.0037	−0.0002	0	0	0	0	0	0	0	0	0	0
**θ2**	0.0000	0.0000	0.0002	−0.0001	0	0	0	0	0	0	0	0	0	0
**W3**	0.0037	0.0002	4.76 × 10^−1^	0.0000	0.0037	−0.0002	0	0	0	0	0	0	0	0
**θ3**	−0.0002	−0.0001	0.0000	0.0000	0.0002	−0.0001	0	0	0	0	0	0	0	0
**W4**	0	0	0.0037	0.0002	0.4761	0.0000	0.0037	−0.0002	0	0	0	0	0	0
**θ4**	0	0	−0.0002	−0.0001	0.0000	0.0000	0.0002	−0.0001	0	0	0	0	0	0
**W5**	0	0	0	0	0.0037	0.0002	2.63 × 10^0^	−0.0004	0	0	0	0	0	0
**θ5**	0	0	0	0	−0.0002	−0.0001	−0.0004	0.0000	0.0002	−0.0001	0	0	0	0
**U6**	0	0	0	0	0	0	0	0.0002	4.76 × 10^−1^	0.0000	0.0037	−0.0002	0	0
**θ6**	0	0	0	0	0	0	0	−0.0001	0.00 × 10^0^	0.0000	0.0002	−0.0001	0	0
**U7**	0	0	0	0	0	0	0	0	0.0037	0.0002	0.4761	0.0000	0.0037	−0.0002
**θ7**	0	0	0	0	0	0	0	0	−0.0002	−0.0001	0.0000	0.0000	0.0002	−0.0001
**U8**	0	0	0	0	0	0	0	0	0	0	0.0037	0.0002	4.66 × 10^−1^	−0.0004
**θ8**	0	0	0	0	0	0	0	0	0	0	−0.0002	−0.0001	−0.0004	0.0000

^1^ Notice state sequence alternates translation, then rotation at each node. 4.76 × 10^−1^ and such indicates $4.76\times 10^{–1}$ , where the former notation is used to ameliorate table crowding.

**Table A3:** Natural frequencies, $\omega_{n}$ [rad/s] ^1^.

1809.5	1415.5	1042.2	774.3	596.8
478.8	410.9	54.9	43.7	30.9
15.8	10.2	0.7	2.1	

^1^ Corresponding to mode shapes in Table 5.

**Table A4:** Normalized mode shapes [×10^4^].

−1	3	2	0	−3	−5	3	1501	383	1037	443	−692	181	240
−1097	3080	4481	4992	4505	3173	−1154	−4544	−136	2388	2221	4049	1395	1669
−1	1	−3	−7	−5	1	−2	−1569	−215	204	667	1425	−670	−712
−2158	4857	3958	28	−3814	−4883	2208	−943	−2040	−7064	−867	912	−2460	−1864
−1	−1	−5	0	7	3	2	1296	−125	−1076	125	995	−1385	−1057
−3111	4495	−1058	−4992	−1185	4481	−3142	5806	2118	368	−2572	4061	−3204	−683
0	0	0	1	1	−1	1	−99	30	113	−105	248	2247	954
−3902	2199	−4861	−47	4875	−2071	3937	−4288	−3426	4504	1878	4753	−3652	1689
0	−3	4	0	−6	6	1	536	−918	135	898	754	−946	736
−4493	−1062	−3138	4985	−3062	−1232	−4519	815	2292	−2423	3091	891	−3893	3998
0	2	5	−7	7	−4	0	−294	753	446	880	410	−1936	1905
−4872	−3903	2129	75	−2232	3920	4832	−2471	3385	−210	−3658	3392	−4013	5178
−1	−2	2	−3	4	−5	−6	90	−261	192	−699	−732	−2945	3254
−5031	−5052	5012	−5030	5062	−4984	−4965	3580	−7823	3941	−7649	−5153	−4046	5502

### Equations of Motion in the Standard State Space Form

(A6)
$${\left\{\begin{array}{c} \dot{x}\\ \ddot{x} \end{array}\right\}}_{\left[nx1\right]}={\left[A\right]}_{nxn}{\left\{\begin{array}{c} x\\ \dot{x} \end{array}\right\}}_{nx1}+{\left[B\right]}_{nx1}{\left\{u\right\}}_{1x1}$$

Finite element analysis performed in MATLAB^®^ (code is included in the appendix) generates the mode shapes used to calculate the rigid–elastic coupling terms. The program outputs the flexible system [A], [B], [C], and [D] matrices of the standard state space form. The results are:

(A7)
$${\left\{\begin{array}{c} \dot{x}\\ \ddot{x} \end{array}\right\}}_{\left[nx1\right]}={\left[\begin{array}{ccc} 0 & 0 & \begin{array}{cc} 1 & 0 \end{array}\\ 0 & 0 & \begin{array}{cc} 0 & 1 \end{array}\\ \begin{array}{c} \frac{{-\omega }_{i}^{2}I_{zz}}{I_{zz}-D_{i}\sum_{i=1}^{n}D_{i}}\\ \frac{{-\omega }_{i}^{2}\sum_{i=1}^{n}D_{i}}{I_{zz}-D_{i}\sum_{i=1}^{n}D_{i}} \end{array} & \begin{array}{c} 0\\ 0 \end{array} & \begin{array}{cc} \begin{array}{c} \frac{-2{{\xi \omega }_{i}I}_{zz}}{I_{zz}-D_{i}\sum_{i=1}^{n}D_{i}}\\ \frac{-2{\xi \omega }_{i}I_{zz}}{I_{zz}-D_{i}\sum_{i=1}^{n}D_{i}} \end{array} & \begin{array}{c} 0\\ 0 \end{array} \end{array} \end{array}\right]}_{nxn}{\left\{\begin{array}{c} x\\ \dot{x} \end{array}\right\}}_{nx1}+{\left[\begin{array}{c} 0\\ 0\\ \begin{array}{c} \frac{-D_{i}T}{I_{zz}-D_{i}\sum_{i=1}^{n}D_{i}}\\ \frac{T}{I_{zz}-D_{i}\sum_{i=1}^{n}D_{i}} \end{array} \end{array}\right]}_{nx1}{\left\{u\right\}}_{1x1}$$

(A8)
$$\left[C\right]=\left[\begin{array}{ccc} 1 & 0 & \begin{array}{ccc} 0 & 0 & \begin{array}{ccc} 0 & 0 & \begin{array}{ccc} 0 & 0 & \begin{array}{ccc} 0 & 0 & \begin{array}{cc} 0 & 0 \end{array} \end{array} \end{array} \end{array} \end{array} \end{array}\right]\;\left[D\right]=\left[0\right]$$

Given these equations, the resultant state space matrices are:

**Table A5:** State space [A] matrix ^1^.

	1	2	3	4	5	6	7	8	9	10	11	12
1	0	0	0	0	0	0	1	0	0	0	0	0
2	0	0	0	0	0	0	0	1	0	0	0	0
3	0	0	0	0	0	0	0	0	1	0	0	0
4	0	0	0	0	0	0	0	0	0	1	0	0
5	0	0	0	0	0	0	0	0	0	0	1	0
6	0	0	0	0	0	0	0	0	0	0	0	1
7	0	0.099	−1.064	−3.382	−0.736	−26.635	0	1.392 × 10^−4^	−5.066 × 10^−4^	−3.298 × 10^−4^	−4.662 × 10^−5^	−8.609 × 10^−4^
8	0	−0.659	1.642	5.218	1.136	41.100	0	−9.264 × 10^−4^	7.817 × 10^−4^	5.088 × 10^−4^	7.194 × 10^−5^	1.328 × 10^−3^
9	0	0.188	−6.435	−6.433	−1.401	−50.670	0	2.649 × 10^−4^	−3.064 × 10^−3^	−6.273 × 10^−4^	−8.869 × 10^−5^	−1.638 × 10^−3^
10	0	0.025	−0.270	−106.024	−0.187	−6.755	0	3.531 × 10^−5^	−1.285 × 10^−4^	−1.034 × 10^−2^	−1.182 × 10^−5^	−2.183 × 10^−4^
11	0	0.002	−0.025	−0.079	−249.447	−0.620	0	3.241 × 10^−6^	−1.179 × 10^−5^	−7.677 × 10^−6^	−1.579 × 10^−2^	−2.004 × 10^−5^
12	0	0.022	−0.233	−0.742	−0.162	−962.998	0	3.055 × 10^−5^	−1.112 × 10^−4^	−7.237 × 10^−5^	−1.023 × 10^−5^	−3.113 × 10^−2^

^1^ Flexible states where base (rigid-body) rotation is controlled. 4.76 × 10^−1^ and such indicates $4.76\times 10^{–1}$ , where the former notation is used to ameliorate table crowding.

(A9)
$$\left[B\right]={\left\{\begin{array}{ccc} 0 & 0 & \begin{array}{ccc} 0 & 0 & \begin{array}{ccc} 0 & 0 & \begin{array}{ccc} 0.126794 & -0.19565 & \begin{array}{ccc} 0.126794 & 0.032156 & \begin{array}{cc} 0.002952 & 0.27828 \end{array} \end{array} \end{array} \end{array} \end{array} \end{array}\right\}}^{T}$$

(A10)
$$\left[C\right]=\left\{\begin{array}{ccc} 1 & 0 & \begin{array}{ccc} 0 & 0 & \begin{array}{ccc} 0 & 0 & \begin{array}{ccc} 0 & 0 & \begin{array}{ccc} 0 & 0 & \begin{array}{cc} 0 & 0 \end{array} \end{array} \end{array} \end{array} \end{array} \end{array}\right\}\;\left[D\right]=\left[0\right]$$

## Appendix B. Initialization Function Callbacks for Simulation

This appendix is an optional section containing details supplemental to the main text crucial to understanding and reproducing the research.

clear all; close all; clc; %This block of code establishes the properties of each beam element a=0.0254; b=0.0016; L=0.25; E=72*10^9; I=a*b^3/12; Li=[12 6*L -12 6*L;6*L 4*L^2 -6*L 2*L^2;-12 -6*L 12 -6*L;6*L 2*L^2 -6*L 4*L^2]; k_beam=E*I/L^3*[Li]; rho_beam=2.8*10^3; %Beam density kg/m^3 A_beam=a*b; %Beam cross sectional area mb=rho_beam*A_beam; %Beam mass per unit length %This block creates the empty stiffness matrix [k] k=zeros(14,14); % This block fills in the stiffness matrix components % Row 1 components start at index=1 % Row 2 components start at index=15 k(1,1)=k_beam(3,3)+k_beam(1,1); k(2,1)=k_beam(4,3)+k_beam(2,1); k(1,2)=k_beam(3,4)+k_beam(1,2); k(2,2)=k_beam(4,4)+k_beam(2,2); k(1,3)=k_beam(1,3); k(2,3)=k_beam(2,3); k(1,4)=k_beam(1,4); k(2,4)=k_beam(2,4); % Row 3 components start at index=29 % Row 4 components start at index=43 k(3,1)=k_beam(3,1); k(4,1)=k_beam(4,1); k(3,2)=k_beam(3,2); k(4,2)=k_beam(4,2); k(3,3)=k_beam(3,3)+k_beam(1,1); k(4,3)=k_beam(4,3)+k_beam(2,1); k(3,4)=k_beam(3,4)+k_beam(1,2); k(4,4)=k_beam(4,4)+k_beam(2,2); k(3,5)=k_beam(1,3); k(4,5)=k_beam(2,3); k(3,6)=k_beam(1,4); k(4,6)=k_beam(2,4); % Row 5 components start at index=59 % Row 6 components start at index=73 k(5,3)=k_beam(3,1); k(6,3)=k_beam(4,1); k(5,4)=k_beam(3,2); k(6,4)=k_beam(4,2); k(5,5)=k_beam(3,3)+k_beam(1,1); k(6,5)=k_beam(4,3)+k_beam(2,1); k(5,6)=k_beam(3,4)+k_beam(1,2); k(6,6)=k_beam(4,4)+k_beam(2,2); k(5,7)=k_beam(1,3); k(6,7)=k_beam(2,3); k(5,8)=k_beam(1,4); k(6,8)=k_beam(2,4); % Row 7 components start at index=89 % Row 8 components start at index=103 k(7,5)=k_beam(3,1); k(8,5)=k_beam(4,1); k(7,6)=k_beam(3,2); k(8,6)=k_beam(4,2); k(7,7)=k_beam(3,3); k(8,7)=k_beam(4,3); k(7,8)=k_beam(3,4); k(8,8)=k_beam(4,4)+k_beam(2,2); k(8,9)=k_beam(2,3); k(8,10)=k_beam(2,4); % Row 9 components start at index=120 % Row 10 components start at index=134 k(9,8)=k_beam(3,2); k(10,8)=k_beam(4,2); k(9,9)=k_beam(3,3)+k_beam(1,1); k(10,9)=k_beam(4,3)+k_beam(2,1); k(9,10)=k_beam(3,4)+k_beam(1,2); k(10,10)=k_beam(4,4)+k_beam(2,2); k(9,11)=k_beam(1,3); k(10,11)=k_beam(2,3); k(9,12)=k_beam(1,4); k(10,12)=k_beam(2,4); % Row 11 components start at index=149 % Row 12 components start at index=163 k(11,9)=k_beam(3,1); k(12,9)=k_beam(4,1); k(11,10)=k_beam(3,2); k(12,10)=k_beam(4,2); k(11,11)=k_beam(3,3)+k_beam(1,1); k(12,11)=k_beam(4,3)+k_beam(2,1); k(11,12)=k_beam(3,4)+k_beam(1,2); k(12,12)=k_beam(4,4)+k_beam(2,2); k(11,13)=k_beam(1,3); k(12,13)=k_beam(2,3); k(11,14)=k_beam(1,4); k(12,14)=k_beam(2,4); % Row 13 components start at index=179 % Row 14 components start at index=193 k(13,11)=k_beam(3,1); k(14,11)=k_beam(4,1); k(13,12)=k_beam(3,2); k(14,12)=k_beam(4,2); k(13,13)=k_beam(3,3); k(14,13)=k_beam(4,3); k(13,14)=k_beam(3,4); k(14,14)=k_beam(4,4); %Display stiffness matrix to check k=k; %END STIFFNESS MATRIX. START MASS MATRIX %Assemble individual beam inertia matrix I_beam=ones(1,8); %Creates empty matrix of I’s for eight node points I_beam=[I_beam.*I]; %Fill in matrix values with beam inertia I_beam(1)=0; %First node point inertia = 0 %This block of code creates the individual beam mass matrix “m_beam” mi=[156 22*L 54 -13*L;22*L 4*L^2 13*L -3*L^2;54 13*L 156 -22*L;-13*L -3*L^2 -22*L 4*L^2]; m_beam=mb*L/420*mi; %This block of code establishes the value of each point mass (mp) %and the system point mass matrix (M) mp=0.455; %Point masses, M M=[0 mp mp mp 2*mp mp mp mp]; %Matrix of 8 point masses (0 First point mass) %Creates a 14x14 empty mass matrix [m] m=zeros(14,14); %Fill in the system mass matrix components % Row 1 components start at index=1 % Row 2 components start at index=15 m(1,1)=m_beam(3,3)+m_beam(1,1)+M(2); m(2,1)=m_beam(4,3)+m_beam(2,1); m(1,2)=m_beam(3,4)+m_beam(1,2); m(2,2)=m_beam(4,4)+m_beam(2,2); m(1,3)=m_beam(1,3); m(2,3)=m_beam(2,3); m(1,4)=m_beam(1,4); m(2,4)=m_beam(2,4); % Row 3 components start at index=29 % Row 4 components start at index=43 m(3,1)=m_beam(3,1); m(4,1)=m_beam(4,1); m(3,2)=m_beam(3,2); m(4,2)=m_beam(4,2); m(3,3)=m_beam(3,3)+m_beam(1,1)+M(3); m(4,3)=m_beam(4,3)+m_beam(2,1); m(3,4)=m_beam(3,4)+m_beam(1,2); m(4,4)=m_beam(4,4)+m_beam(2,2); m(3,5)=m_beam(1,3); m(4,5)=m_beam(2,3); m(3,6)=m_beam(1,4); m(4,6)=m_beam(2,4); % Row 5 components start at index=59 % Row 6 components start at index=73 m(5,3)=m_beam(3,1); m(6,3)=m_beam(4,1); m(5,4)=m_beam(3,2); m(6,4)=m_beam(4,2); m(5,5)=m_beam(3,3)+m_beam(1,1)+M(4); m(6,5)=m_beam(4,3)+m_beam(2,1); m(5,6)=m_beam(3,4)+m_beam(1,2); m(6,6)=m_beam(4,4)+m_beam(2,2); m(5,7)=m_beam(1,3); m(6,7)=m_beam(2,3); m(5,8)=m_beam(1,4); m(6,8)=m_beam(2,4); % Row 7 components start at index=89 m(7,5)=m_beam(3,1); m(7,6)=m_beam(3,2); m(7,7)=m_beam(3,3)+3*mb+M(5)+M(6)+M(7)+M(8); m(7,8)=m_beam(3,4); % Row 8 components start at index=103 % Row 9 components start at index=120 m(8,5)=m_beam(4,1); m(9,8)=m_beam(3,2); m(8,6)=m_beam(4,2); m(9,9)=m_beam(3,3)+m_beam(1,1)+M(6); m(8,7)=m_beam(4,3); m(9,10)=m_beam(3,4)+m_beam(1,2); m(8,8)=m_beam(4,4)+m_beam(2,2); m(9,11)=m_beam(1,3); m(8,9)=m_beam(2,3); m(9,12)=m_beam(1,4); m(8,10)=m_beam(2,4); % Row 10 components start at index=134 % Row 11 components start at index=149 m(10,8)=m_beam(4,2); m(11,9)=m_beam(3,1); m(10,9)=m_beam(4,3)+m_beam(2,1); m(11,10)=m_beam(3,2); m(10,10)=m_beam(4,4)+m_beam(2,2); m(11,11)=m_beam(3,3)+m_beam(1,1)+M(7); m(10,11)=m_beam(2,3); m(11,12)=m_beam(3,4)+m_beam(1,2); m(10,12)=m_beam(2,4); m(11,13)=m_beam(1,3); m(11,14)=m_beam(1,4); % Row 12 components start at index=163 m(12,9)=m_beam(4,1); m(12,10)=m_beam(4,2); m(12,11)=m_beam(4,3)+m_beam(2,1); m(12,12)=m_beam(4,4)+m_beam(2,2); m(12,13)=m_beam(2,3); m(12,14)=m_beam(2,4); % Row 13 components start at index=179 % Row 14 components start at index=193 m(13,11)=m_beam(3,1); m(14,11)=m_beam(4,1); m(13,12)=m_beam(3,2); m(14,12)=m_beam(4,2); m(13,13)=m_beam(3,3)+M(8); m(14,13)=m_beam(4,3); m(13,14)=m_beam(3,4); m(14,14)=m_beam(4,4); %Display the system mass matrix to check m=m; %Calculate the natural frequencies and normal modes [NormalModes,EigenValues]=eig(inv(m)*k); NaturalFrequencies=diag(EigenValues^0.5); ModeShapes=NormalModes; %Check Orthogonality like Homework 1 confirm diagonal matrix of 1’s %to satisfy equation 24 on slide 17 OrthoMass=NormalModes’*m*NormalModes; OrthoStiff=NormalModes’*k*NormalModes; StiffCheck=OrthoStiff/EigenValues; Equation24_OrthoCheck=diag(diag(StiffCheck/OrthoMass)); %Spacecraft Radius to be used designating rigid modal coordinate R=0.381; FeeE=NormalModes; %Designate Elastic mode shapes array FeeE Omega=NaturalFrequencies; %Designate variable name ‘Omega’ as natural frequencies %Designate Rigid modal coordinate FeeR FeeR=[R+L 1 R+L*2 1 R+L*3 1 R+L*4 1 -L 1 -L*2 1 -L*3 1]; Di=FeeE’*m*diag(FeeR); %Calculate Rigid-Elastic Coupling Coefficient DiCheck=det(Di); %Confirm Di is singular...det(Di=0) Z=0.0005; Izz=14; w=diag(NaturalFrequencies); %Generate a diagonal matrix of natural frequency Iw=0.0912; Td=0; %Disturbance Torque Tc=0.1; %Control Torque is Iw*qddot_wheel T=Td+Tc; %Total Torque is sum of disturbance and control torques %Start State Space Development NatFreq = diag(EigenValues).^0.5; r = 0.381; %Radius of the wheel (large rigid body) freqs = sqrt(EigenValues); % NatFreq = EigenValues(1:5,1:5); freqs = freqs(1:5,1:5); zeta = 0.0005; %Given damping ratio for all modes Izz = 14; phi_E = NormalModes(1:14,1:5); phi_R = [r+L,1,r+2*L,1,r+3*L,1,r+4*L,1,-L,1,-2*L,1,-3*L,1]’; M_II = m; Di = [phi_E’*M_II*phi_R]; M_state = [Izz Di’; Di eye(5)]; C_damp = [zeros(6,6)]; C_damp(2:6,2:6) = 2*zeta*freqs; K=[zeros(6,6)]; K(2:6,2:6) = NatFreq; A = [zeros(6),eye(6,6); -inv(M_state)*K, -inv(M_state)*C_damp]; Bprime = [1;0;0;0;0;0]; B = [0 0 0 0 0 0 (inv(M_state)*Bprime)’]’; C = zeros(12,12); C(1,1)=1; D = zeros(12,1); [Gnum,Gden] = ss2tf(A,B,C,D); G1 = tf(Gnum(1,:),Gden) %Manually input Transfer Function to check NUM=[1.998e-015 0.1268 0.007582 166.9 5.591 4.718e004 771 3.412e006 1.218e004 1.576e007 1.475e004 7.11e006]; DEN=[1 0.06125 1326 46.15 3.781e005 6683 2.808e007 1.388e005 1.813e008 2.065e005 9.954e007 0 0]; G=tf(NUM,DEN); %Put PID controller Transfer function into workspace It=14; Z=0.516931; Bandwidth=4; wn=Bandwidth; T=10/Z/wn; Kd=2*Z*wn*It+It/T; Kp=wn^2+2*Z*wn/T; Ki=wn^2/T; PID=tf([Kd Kp Ki],[0 1 0]); %DESIGN FILTERS TO SMOOTH OUT MODE 1 %Design Bandpass filter for w = 10^-0.1478 = 0.711541 Hz wz=0.711541;Zz=0.1;wp=wz;Zp=0.0005; BP1=tf([1/wz^2 2*Zz/wz 1],[1/wp^2 2*Zp/wp 1]); PID_BP1=PID*BP1; %Design Notch filter for w = 10^-0.109 = 0.778037 Hz wz=0.778037;Zz=0.0005;wp=wz;Zp=0.1; Notch1=tf([1/wz^2 2*Zz/wz 1],[1/wp^2 2*Zp/wp 1]); Mode_1=PID*BP1*Notch1; %DESIGN FILTERS TO SMOOTH OUT MODE 2 %Design Bandpass filter for w = 10^0.3223 wz=10^0.3223;Zz=0.1;wp=wz;Zp=0.0005; BP2=tf([1/wz^2 2*Zz/wz 1],[1/wp^2 2*Zp/wp 1]); %Design Notch filter for w = 10^0.405 wz=10^0.405;Zz=0.0006;wp=wz;Zp=0.1; Notch2=tf([1/wz^2 2*Zz/wz 1],[1/wp^2 2*Zp/wp 1]); Mode_2=Mode_1*BP2*Notch2; %Design Lead filter for wz~1, wp~3 %wz=1;Zz=1;wp=3;Zp=1; %Lead=tf([1/wz^2 2*Zz/wz 1],[1/wp^2 2*Zp/wp 1]); %Mode_2=Mode_2*Lead; %DESIGN FILTERS TO SMOOTH OUT MODE 3 %Design Bandpass filter for w = 10^1.0110 wz=10^1.0110;Zz=0.1;wp=wz;Zp=0.0005; BP3=tf([1/wz^2 2*Zz/wz 1],[1/wp^2 2*Zp/wp 1]); %Design Notch filter for w = 10^1.0128 wz=10^1.0128;Zz=0.0005;wp=wz;Zp=0.1; Notch3=tf([1/wz^2 2*Zz/wz 1],[1/wp^2 2*Zp/wp 1]); Mode_3=Mode_2*BP3*Notch3; %DESIGN FILTERS TO SMOOTH OUT MODE 4 %Design Bandpass filter for w = 10^1.49035 wz=10^1.49035;Zz=0.1;wp=wz;Zp=0.0005; BP4=tf([1/wz^2 2*Zz/wz 1],[1/wp^2 2*Zp/wp 1]); %Design Notch filter for w = 10^1.492 wz=10^1.492;Zz=0.0005;wp=wz;Zp=0.1; Notch4=tf([1/wz^2 2*Zz/wz 1],[1/wp^2 2*Zp/wp 1]); Mode_4=Mode_3*BP4*Notch4; %CALCULATE SYSTEM NATURAL FREQUENCIES [NaturalFrequencies,Damping,EigenValue]=damp(G); NaturalFrequencies=NaturalFrequencies;

## Appendix C. Stop Function Callbacks for Simulation

This appendix is an optional section containing details supplemental to the main text crucial to understanding and reproducing the research.

[mag1,phase1,wout1] = bode(G); Mag1=20*log10(mag1(:)); Phase1=phase1(:); [mag2,phase2,wout2] = bode(G*PID); Mag2=20*log10(mag2(:)); Phase2=phase2(:); [mag3,phase3,wout3] = bode(G*PID*BP1); Mag3=20*log10(mag3(:)); Phase3=phase3(:); [mag4,phase4,wout4] = bode(G*PID*BP1*Notch1); Mag4=20*log10(mag4(:)); Phase4=phase4(:); [mag5,phase5,wout5] = bode(G*PID*BP1*Notch1*BP2); Mag5=20*log10(mag5(:)); Phase5=phase5(:); [mag6,phase6,wout6] = bode(G*PID*BP1*Notch1*BP2*Notch2); Mag6=20*log10(mag6(:)); Phase6=phase6(:); [mag7,phase7,wout7] = bode(G*PID*BP1*Notch1*BP2*Notch2*BP3); Mag7=20*log10(mag7(:)); Phase7=phase7(:); [mag8,phase8,wout8] = bode(G*PID*BP1*Notch1*BP2*Notch2*BP3*Notch3); Mag8=20*log10(mag8(:)); Phase8=phase8(:); [mag9,phase9,wout9] = bode(G*PID*BP1*Notch1*BP2*Notch2*BP3*Notch3*BP4); Mag9=20*log10(mag9(:)); Phase9=phase9(:); [mag10,phase10,wout10] = bode(G*PID*BP1*Notch1*BP2*Notch2*BP3*Notch3*BP4*Notch4); Mag10=20*log10(mag10(:)); Phase10=phase10(:); figure(1); hold on; semilogx(wout1,Mag1,‘--’,‘LineWidth’,1); semilogx(wout2,Mag2,‘LineWidth’,1); semilogx(wout3,Mag3,‘--’,‘LineWidth’,3); semilogx(wout4,Mag4,‘:’,‘LineWidth’,2); hold off; grid on; axis([0.5,40, -100, 150 ]); set(gca, ‘FontSize’,28, ‘FontName’,‘Palatino Linotype’); legend(‘Flexible space robot’,‘PID’,‘PID + Bandpass’,‘PID + Notch + Bandpass’) figure(2); hold on; set(gca, ‘FontSize’,28, ‘FontName’,‘Palatino Linotype’); semilogx(wout1,Phase1,’--’,‘LineWidth’,1); semilogx(wout2,Phase2,’LineWidth’,1); semilogx(wout3,Phase3,’--’,‘LineWidth’,3); semilogx(wout4,Phase4,’:’,‘LineWidth’,2); hold off; grid on; figure(3); hold on; semilogx(wout2,Mag2,’--’,‘LineWidth’,1); semilogx(wout4,Mag4,’LineWidth’,1); semilogx(wout5,Mag5,’--’,‘LineWidth’,3); semilogx(wout6,Mag6,’:’,‘LineWidth’,2); hold off; grid on; axis([0.5,40, -100, 150 ]); set(gca, ‘FontSize’,28, ‘FontName’,‘Palatino Linotype’); legend(‘PID controlled Flexible space robot’,‘PID+Mode 1’,‘PID + Mode 1 + Bandpass’,‘PID + Mode 1 + Notch + Bandpass’) figure(4); hold on; set(gca, ‘FontSize’,28, ‘FontName’,‘Palatino Linotype’); semilogx(wout2,Phase2,’--’,‘LineWidth’,1); semilogx(wout4,Phase4,’LineWidth’,1); semilogx(wout5,Phase5,’--’,‘LineWidth’,3); semilogx(wout6,Phase6,’:’,‘LineWidth’,2); hold off; grid on; figure(5); hold on; semilogx(wout2,Mag2,’--’,‘LineWidth’,1); semilogx(wout4,Mag4,’LineWidth’,1); semilogx(wout6,Mag6,’--’,‘LineWidth’,3); semilogx(wout7,Mag7,’:’,‘LineWidth’,2); semilogx(wout8,Mag8,’:’,‘LineWidth’,2); hold off; grid on; axis([0.5,40, -100, 150 ]); set(gca, ‘FontSize’,28, ‘FontName’,‘Palatino Linotype’); legend(‘PID controlled Flexible space robot’,‘PID + Mode 1’,‘PID + Mode 2’,‘PID + Mode 1 + Mode 2 + Bandpass’,‘PID + Mode 1 + Mode 2 + Bandpass + Notch’) figure(6); hold on; set(gca, ‘FontSize’,28, ‘FontName’,‘Palatino Linotype’); semilogx(wout2,Phase2,’--’,‘LineWidth’,1); semilogx(wout4,Phase4,’LineWidth’,1); semilogx(wout6,Phase6,’--’,‘LineWidth’,3); semilogx(wout7,Phase7,’:’,‘LineWidth’,2); semilogx(wout8,Phase8,’:’,‘LineWidth’,2); hold off; grid on; figure(7); hold on; semilogx(wout2,Mag2,’--’,‘LineWidth’,1); semilogx(wout4,Mag4,’LineWidth’,1); semilogx(wout6,Mag6,’--’,‘LineWidth’,3); semilogx(wout8,Mag8,’:’,‘LineWidth’,2); semilogx(wout9,Mag9,’:’,‘LineWidth’,2); semilogx(wout10,Mag10,’:’,‘LineWidth’,2); hold off; grid on; axis([0.5,40, -100, 150 ]); set(gca, ‘FontSize’,28, ‘FontName’,‘Palatino Linotype’); legend(‘PID controlled Flexible space robot’,‘PID + Mode 1’,‘PID + Mode 2’,‘PID + Mode 1 + Mode 2 + Mode 3’,‘PID + Mode 1 + Mode 2 + Mode 3 + Bandpass + Notch’) figure(8); hold on; set(gca, ‘FontSize’,28, ‘FontName’,‘Palatino Linotype’); semilogx(wout2,Phase2,’--’,‘LineWidth’,1); semilogx(wout4,Phase4,’LineWidth’,1); semilogx(wout6,Phase6,’--’,‘LineWidth’,3); semilogx(wout8,Phase8,’:’,‘LineWidth’,2); semilogx(wout9,Phase9,’:’,‘LineWidth’,2); semilogx(wout10,Phase10,’:’,‘LineWidth’,2); hold off; grid on; sys1=G*PID/(1+G*PID); sys2=(G*PID*BP1/(1+G*PID*BP1)); sys3=(G*PID*BP1*Notch1/(1+G*PID*BP1*Notch1)); sys4=(G*PID*BP1*Notch1*BP2/(1+G*PID*BP1*Notch1*BP2)); sys5=(G*PID*BP1*Notch1*BP2*Notch2/(1+G*PID*BP1*Notch1*BP2*Notch2)); sys6=(G*PID*BP1*Notch1*BP2*Notch2*BP3/(1+G*PID*BP1*Notch1*BP2*Notch2*BP3)); sys7=(G*PID*BP1*Notch1*BP2*Notch2*BP3*Notch3/(1+G*PID*BP1*Notch1*BP2*Notch2*BP3*Notch3)); sys8=(G*PID*BP1*Notch1*BP2*Notch2*BP3*Notch3*BP4/(1+G*PID*BP1*Notch1*BP2*Notch2*BP3*Notch3*BP4)); sys9=(G*PID*BP1*Notch1*BP2*Notch2*BP3*Notch3*BP4*Notch4/(1+G*PID*BP1*Notch1*BP2*Notch2*BP3*Notch3*BP4*Notch4)); figure(9); step(sys1,sys2); legend(‘PID’,‘PID + BP1’);set(gca, ‘FontSize’,28, ‘FontName’,‘Palatino Linotype’); figure(10); step(sys1,sys3); legend(‘PID’,‘PID + BP1+Notch1’); set(gca, ‘FontSize’,28, ‘FontName’,‘Palatino Linotype’); figure(11); step(sys1,sys4); legend(‘PID’,‘PID + Mode 1 + BP2’); set(gca, ‘FontSize’,28, ‘FontName’,‘Palatino Linotype’); figure(12); step(sys1,sys5); legend(‘PID’,‘PID + Mode 1 + BP2 + Notch 2’); set(gca, ‘FontSize’,28, ‘FontName’,‘Palatino Linotype’); figure(13); step(sys1,sys6); legend(‘PID’,‘PID + Mode 1 + Mode 2 + BP3’); set(gca, ‘FontSize’,28, ‘FontName’,‘Palatino Linotype’); figure(14); step(sys1,sys7); legend(‘PID’,‘PID + Mode 1 + Mode 2 + BP3 + Notch3’); set(gca, ‘FontSize’,28, ‘FontName’,‘Palatino Linotype’); figure(15); step(sys1,sys8); legend(‘PID’,‘PID + Mode 1 + Mode 2 + Mode 3 + BP4’); set(gca, ‘FontSize’,28, ‘FontName’,‘Palatino Linotype’); figure(16); step(sys1,sys9); legend(‘PID’,‘PID + Mode 1 + Mode 2 + Mode 3 + BP4 + Notch4’); set(gca, ‘FontSize’,28, ‘FontName’,‘Palatino Linotype’);
